# Dietary Nitrate Supplementation and Exercise Performance: An Umbrella Review of 20 Published Systematic Reviews with Meta-analyses

**DOI:** 10.1007/s40279-025-02194-6

**Published:** 2025-03-14

**Authors:** Eric Tsz-Chun Poon, Jason Chun-Kit Iu, Wesley Man-Kuk Sum, Po-San Wong, Kenneth Ka-Hei Lo, Ajmol Ali, Stephen F. Burns, Eric T. Trexler

**Affiliations:** 1 Department of Sports Science and Physical Education, The Chinese University of Hong Kong, Shatin, Hong Kong; 2 Department of Education, Hong Kong College of Technology, Sha Tin, Hong Kong; 3 Department of Food Science and Nutrition, The Hong Kong Polytechnic University, Hung Hom, Hong Kong; 4 School of Sport, Exercise and Nutrition, Massey University, Auckland, 0745 New Zealand; 5 Physical Education and Sports Science, National Institute of Education, Nanyang Technological University, 1 Nanyang Walk, Singapore, 637616 Singapore; 6 Department of Evolutionary Anthropology, Duke University, Durham, NC USA

## Abstract

**Background:**

Dietary nitrate (NO_3_^−^) supplementation is purported to benefit exercise performance. However, previous studies have evaluated this nutritional strategy with various performance outcomes, exercise tasks, and dosing regimens, often yielding inconsistent results that limit the generalizability of the findings.

**Objective:**

We aimed to synthesize the available evidence regarding the effect of NO_3_^−^ supplementation on 11 domains of exercise performance.

**Methods:**

An umbrella review was reported in accordance with the Preferred Reporting Items for Overviews of Reviews guideline. Seven databases (MEDLINE, EMBASE, Cochrane Database, CINAHL, Scopus, SPORTDiscus, and Web of Science) were searched from inception until July 2024. Systematic reviews with meta-analyses comparing NO_3_^−^ supplementation and placebo-controlled conditions were included. Literature search, data extraction, and methodological quality assessment (A Measurement Tool to Assess Systematic Reviews Assessing the Methodological quality of SysTemAtic Review [AMSTAR-2]) were conducted independently by two reviewers.

**Results:**

Twenty systematic reviews with meta-analyses, representing 180 primary studies and 2672 unique participants, met the inclusion criteria. Our meta-analyses revealed mixed effects of NO_3_^−^ supplementation. It improved time-to-exhaustion tasks [standardized mean difference (SMD): 0.33; 95% confidence interval (CI) 0.19–0.47] with subgroup analyses indicating more pronounced improvements when a minimum dose of 6 mmoL/day (372 mg/day) and chronic (> 3 days) supplementation protocol was implemented. Additionally, ergogenic effects of NO_3_^−^ supplementation were observed for total distance covered (SMD: 0.42; 95% CI 0.09–0.76), muscular endurance (SMD: 0.48; 95% CI 0.23–0.74), peak power output (PPO; SMD: 0.25; 95% CI 0.10 to 0.39), and time to PPO (SMD: − 0.76; 95% CI − 1.18, − 0.33). However, no significant improvements were found for other performance outcomes (all *p* > 0.05). The AMSTAR-2 ratings of most included reviews ranged from low to critically low.

**Conclusions:**

This novel umbrella review with a large-scale meta-analysis provides an updated synthesis of evidence on the effects of NO_3_^−^ supplementation across various aspects of exercise performance. Our review also highlights significant methodological quality issues that future systematic reviews in this field should address to enhance the reliability of evidence.

**Clinical Trial Registration:**

This study was registered in the International Prospective Register of Systematic Review (PROSPERO) database (registration number: CRD42024577461).

**Supplementary Information:**

The online version contains supplementary material available at 10.1007/s40279-025-02194-6.

## Key Points

**Table:** 

Previous reviews have evaluated dietary nitrate supplementation with diverse performance outcomes, exercise tasks, and dosing regimens, posing challenges for nutrition and exercise professionals to interpret the body of evidence regarding its effects and applications.
Our umbrella review indicated that nitrate supplementation improves time-to-exhaustion tasks, total distance covered, muscular endurance, peak power output, and time to peak power output, but does not demonstrate ergogenic effects on other performance outcomes.
Our review also highlights significant methodological quality issues that future systematic reviews in this field should address to enhance the reliability of evidence.

## Introduction

Nitric oxide (NO) is a crucial signaling and regulatory molecule involved in various physiological processes such as vasodilation, angiogenesis, mitochondrial respiration, muscle glucose uptake, and sarcoplasmic reticulum calcium handling [1]. The human body has two complementary pathways to generate NO: the NO synthase-dependent pathway (i.e., the biosynthesis of NO from the conversion of L-arginine to L-citrulline in the presence of oxygen) and the nitrate-nitrite-NO pathway, which requires a series of intricate inter-organ reactions [2, 3]. The latter pathway is fueled by dietary consumption of nitrate (NO_3_^−^)-rich foods, such as green leafy or root vegetables, which account for ~ 80% of the body’s NO_3_^−^ supply [4, 5]. Ingested NO_3_^−^ is then converted to nitrite by anaerobic bacteria present in the oral cavity, which can be further reduced to NO, particularly under conditions of hypoxia or acidosis [2, 3]. Given the unique role of NO in improving mitochondrial and muscle contractile efficiency during exercise [6, 7], NO_3_^−^ consumption in the form of high-nitrate-containing foods or juice, such as beetroot, spinach, kale, and carrots, has been extensively studied for its potential benefits on exercise performance over the past two decades [8, 9]. In 2018, The International Olympic Committee published a consensus statement [10] addressing the effect of dietary supplements on athletic performance, suggesting that NO_3_^−^ supplementation is associated with improvements in prolonged submaximal exercise and high-intensity intermittent, team-sport exercise of 12–40 min in duration. However, the performance impacts of NO_3_^−^ underlying this statement were primarily based on a limited number of original studies available at the time of publication (2018) [11–14]. More recent evidence suggests that the performance enhancement benefits with NO_3_^−^ ingestion appear most beneficial for exercise lasting 2–10 min [15]. Additionally, a recently published expert consensus, derived through the modified Delphi technique, has provided further insights into potential modifiers of the ergogenic effects of NO_3_^−^ supplementation [16]. Despite ongoing advancements in this field, the expert consensus identified several key limitations in the current literature, including small sample sizes and a narrow focus on specific exercise tasks or experimental conditions [16]. This underscores the need for future research to employ more novel and robust study designs to advance this area of inquiry.

In the field of sports nutrition, systematic reviews and meta-analyses synthesize the available primary studies to reflect the quantity and quality of research available based on the inclusion or exclusion criteria needed to answer specific questions [10]. Nevertheless, existing systematic reviews and meta-analyses on NO_3_^−^ supplementation and exercise performance have often adopted a singular focus on one specific performance domain, such as cardiorespiratory endurance [8, 17–21], muscular strength [22–26], or high-intensity power output parameters [27, 28]. This narrow approach may overlook the broader implications and benefits of NO_2_^−^ across various types of performance outcomes. For instance, in sports that involve different types of exercise, such as team sports with prolonged activity and brief anaerobic or sprinting periods, generalized information may be needed to capture these diverse contexts. Furthermore, individual reviews have varied in sub-population groups (e.g., healthy populations or well-trained athletes), dosing regimens (e.g., acute or chronic supplementation protocols of various doses), or exercise task types, often leading to conflicting findings. For example, while some individual systematic reviews have shown NO_3_^−^ supplementation to be effective in improving exercise performance compared with placebo [22, 24, 26, 29, 30], others suggest no significant advantage [23, 31]. This heterogeneity and discrepancies in the evidence pose challenges for nutrition and exercise professionals seeking to interpret the body of evidence regarding the impact of NO_3_^−^ supplementation on various performance outcomes.

Umbrella reviews, also known as overviews of reviews or meta-reviews, have been proposed as a strategy to comprehensively synthesize evidence on a given topic [32]. Umbrella reviews summarize existing evidence from systematic reviews and may provide an even more reliable and comprehensive foundation for informing evidence-based guidelines compared with individual systematic reviews [32]. They also encompass a broader time frame, as some systematic reviews and meta-analyses are limited to specific years of study [32]. Their ability to synthesize the totality of systematic review-level evidence makes them an invaluable resource for researchers, sports nutritionists, coaches, and athletes. To the best of our knowledge, no umbrella review has been conducted to date on NO_3_^−^ supplementation and exercise performance. Considering the substantial increase in relevant evidence published through systematic reviews and meta-analyses in recent years, an umbrella review addressing the aforementioned research gaps to further establish the comparative benefits and applications of NO_3_^−^ supplementation across various domains of exercise performance appears timely. Therefore, the primary aim of this review was to undertake the most comprehensive synthesis of evidence to date regarding the effect of NO_3_^−^ supplementation on a broad range of exercise performance outcomes. We also aimed to critically appraise the methodological qualities of existing systematic reviews with meta-analyses in this field to inform future research directions.

## Methods

### Search Strategy

Our umbrella review of systematic reviews with meta-analyses followed the Preferred Reporting Items for Overviews of Reviews (PRIOR) statement [32]. The protocol for the umbrella review was registered in the PROSPERO database (CRD42024577461). The review process began before the registration was finalized and focused exclusively on peer-reviewed systematic review articles published in English from inception until 1 July, 2024. Seven databases (MEDLINE, EMBASE, Cochrane Database, CINAHL, Scopus, SPORTDiscus, and Web of Science) were searched using subject heading, keyword, and Medical Subject Headings term searches for ‘systematic review,’ ‘meta-analysis,’ ‘dietary nitrate,’ and ‘exercise performance’ (a detailed search strategy is presented in Table S1 of the Electronic Supplementary Material [ESM]). The reference lists of the selected review articles were also examined for other potentially eligible papers.

### Selection Procedure and Eligibility Criteria

The population, intervention, comparison, outcomes and study type (PICOS) framework was used to develop the inclusion criteria.

#### Types of Populations

The population of interest was human participants. No exclusion criteria were applied to participants’ age, sex, and baseline fitness. Reviews that solely targeted individuals with specific clinical conditions (e.g., heart failure, coronary artery disease, chronic obstructive pulmonary disease, stroke, spinal cord injuries, diabetes mellitus, or cancers) were excluded. However, reviews that included clinical populations as part of a broader sample were retained to maximize the inclusion of relevant evidence that demonstrates the overall efficacy of NO_3_^–^ supplementation for the general population.

#### Types of Interventions

Any acute studies (defined as a single dose intake within a day) or chronic studies (defined as multiple intakes over an extended period, typically several days to weeks) that examined the effects of NO_3_^–^ on exercise performance were included.

#### Type of Comparator

Reviews that involved placebo-controlled conditions or groups (i.e., without NO_3_^–^ so that its effects could be isolated) were included. Reviews with no comparison conditions or groups, or those comparing with baseline values only, were excluded.

#### Types of Outcomes

The outcome of interest in this umbrella review was any form of exercise performance, including aerobic endurance (i.e., time-to-exhaustion [TTE], time trial [TT], graded exercise tests [GXT], total work done [TWD], total distance covered, and maximal oxygen uptake [$\dot{V}$ O_2_max]), muscular fitness (i.e., muscular strength and muscular endurance), and high-intensity power output performance (i.e., peak power output [PPO], mean power output [MPO], and time to reach PPO).

#### Types of Studies

Systematic reviews with meta-analyses were selected.

### Data Management and Extraction

Search results were imported into EndNote X10 (Clarivate, Philadelphia, PA, UA) where duplicates were removed. Two independent reviewers (EP and JI) conducted title/abstract and full-text screening in duplicate. Inter-reviewer disagreements were resolved by consensus or arbitration by a third reviewer (WS). Data were extracted using a standardized extraction form, and two independent reviewers (EP and JI) performed the data extraction in duplicate. The extracted data included the lead author, year of publication, population characteristics, number of original studies, design of original studies, sample size, major performance outcomes and findings. Discrepancies were resolved through consensus or arbitration by a third reviewer (WS).

### Methodological Quality Assessment of Included Systematic Reviews

Two independent reviewers (EP and JI) assessed the methodological quality of the included reviews in duplicate using AMSTAR-2 (A MeaSurement Tool to Assess systematic Reviews) [33]. Discrepancies were resolved through consensus or arbitration by a third reviewer (WS). The AMSTAR-2 consists of 16 items, each scored as ‘yes,’ ‘partial yes,’ or ‘no’. In this review, six items were considered ‘critical’, and ten were considered ‘non-critical’. The critical domains included protocol registration, adequacy of search strategy, risk of bias (RoB) assessment, appropriateness of meta-analysis methods, use of RoB during interpretation, and assessment of publication bias. Reviews were rated as having ‘high confidence’ (0 or 1 non-critical weakness), ‘moderate’ (> 1 non-critical weakness but 0 critical flaws), ‘low’ (1 critical flaw with or without non-critical weaknesses), or ‘critically low’ (> 1 critical flaw with or without non-critical weaknesses) [33].

### Umbrella Review Synthesis Methods

The overlap in component primary studies included in all eligible reviews was assessed using the Corrected Covered Area (CCA) formula [34]: CCA = (*N − r*)/(*rc − r*), where *N* is the sum of the total primary studies included in all the reviews, *r* is the number of unique primary studies, and *c* is the total number of reviews. The CCA ranges from 0 to 100%, with 100% indicating that all the reviews in an umbrella review included the same component original studies, and 0% indicating that each review included entirely unique original studies. The CCA was categorized based on the following cut-offs: 0–5% as ‘slight’; 6–10% as ‘moderate’; 11–15% as ‘high’; and > 15% as ‘very high’ overlap [34].

Meta-analysis results from each review that the reported standardized effect size (e.g., standardized mean difference [SMD]) and 95% confidence intervals (CIs) were presented using forest plots. Data reported by each review were cross-checked with original data reported by the primary studies for consistency. Aggregated results were summarized using medians and ranges, as performed previously [35, 36].

### Additional Meta-Analyses Based on Primary Studies

To address the potentially high overlap rates between individual reviews, we conducted additional meta-analyses using eligible primary studies (i.e., randomized controlled trials) included in all reviews, as employed in relevant prior research [37]. Our analytical approach aligned with the guidance provided in the *Cochrane Handbook for Systematic Reviews of Interventions* [38]. The absolute change in mean difference and standard deviation of the outcome values from post-intervention between groups in each study was calculated, and pooled using the DerSimonian and Laird random-effects method (RevMan Version 5.4.1; Cochrane Collaboration, Oxford, UK). Standardized mean differences with 95% CIs were used to synthesize continuous outcomes and create forest plots. To address the potential unit-of-analysis error, we followed the *Cochrane Handbook for Systematic Reviews of Interventions* recommendation by combining all relevant experimental intervention groups (e.g., NO_3_^–^ supplementation with varying dosages and durations) and comparator groups (e.g., various placebo solutions without NO_3_^–^) into single groups within individual studies, creating a single pair-wise comparison for the overall analysis [38]. Heterogeneity among studies was assessed using the Chi-square test, while the degree of inconsistency was quantified with the I-square statistic. I-square values of < 25%, 50%, and 75% were considered indicative of low, moderate, and high heterogeneity, respectively [39]. To enhance the robustness of our findings, we performed sensitivity analyses using the leave-one-out method. This approach involves removing one study at a time to evaluate its impact on the overall results and to assess the influence of individual studies on the collective findings. Subgroup analyses were conducted based on the supplementation protocol duration (acute, 1 − 3 days, or > 3 days) and daily dose (< 6 mmol, 6 − 12 mmol, or > 12 mmol) for outcomes with at least three studies in each comparison arm.

## Results

### Overview of Search Results

**Fig. 1:**
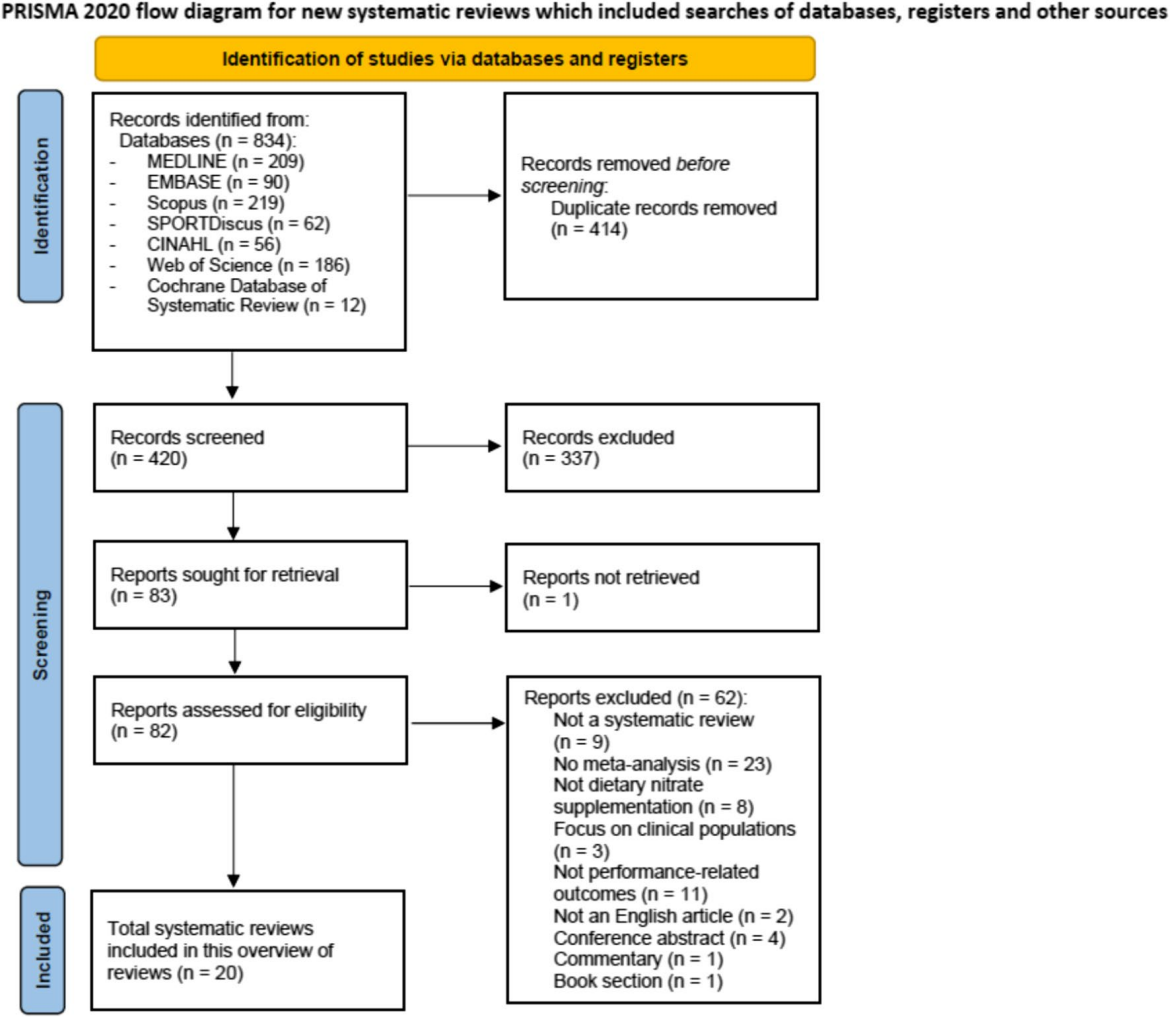
Preferred Reporting Items for Systematic Reviews and Meta-Analysis (PRISMA) 2020 flowchart of literature selection on systematic reviews

The search strategy yielded a total of 834 records from seven electronic databases. After removing duplicates, 420 records remained, out of which 337 were subsequently excluded based on title and abstract screening. The full texts of the remaining 82 articles were assessed, and 20 systematic reviews and meta-analyses that met the inclusion criteria were included in this umbrella review (refer to Fig. 1 for flowchart and reasons for exclusions in Table S2 of the ESM).

### Characteristics of Included Reviews

Table 1 presents a summary of the author, year, study type, participant characteristics, performance outcomes, and main findings of the included systematic reviews. The sample sizes of the 20 systematic reviews ranged from 43 [40] to 1705 [15]. A total of 180 unique primary studies with 2672 unique participants were listed in the included systematic reviews (Table S3 of the ESM), with a CCA of 14.4% indicating a high overlap. The publication year range of the primary studies was from 2007 to 2022. Four systematic reviews [22, 23, 25, 27] consisted solely of a double-blind, randomized crossover design, while other included reviews included both single-blind and double-blind studies or did not account for blinding in the inclusion criteria. Eleven reviews reported on the sex composition of the included participants [17, 19, 20, 23, 25–28, 30, 31, 41], and all of these reviews found a predominance of male over female participants. Notably, most included reviews [15, 17, 18, 20, 21, 24, 25, 28–30, 40–42] (*n* = 13) focused on healthy individuals, one review specifically focused on resistance-trained male adults [22], while one review solely involved elite or well-trained athletes [19]. Additionally, two reviews included individuals with various health statuses [23, 31]. Of note, the three primary studies [43–45] included in these two reviews that focused on clinical populations were excluded in the subsequent meta-analyses to avoid contamination.

**Table 1:** Summary of included systematic reviews

Reference	Included studies and populations	Study design	Sample size	Major performance outcomes	Main findings from individual reviews	GRADE assessment
Alsharif et al. 2023 [29]	*k* = 27 Age: 17–31 years; healthy adults	Single or double-blind, randomized crossover design	Total: 410	TWD, TDC, PPO, MPO, Time to reach PPO	NO_3_^−^ supplementation had small positive effects on some performance outcomes during single and repeated bouts of high-intensity exercise	NA
Alvares et al. 2022 [26]	*k* = 34 Age: no restriction; individuals not hospitalized or critically ill	Randomized crossover or parallel design	Total: 475 (397 male, 78 female)	MS, ME	NO_3_^−^ supplementation seems to have a positive effect on MS and ME, which is mostly unaffected by dosage, frequency of ingestion, training level, muscle group, or type of contraction	NA
Campos et al. 2018 [42]	*k* = 54 Age: not specified; healthy individuals (either non-athletes or athletes)	Placebo-controlled, crossover design	Total: 662	TTE, TT, GXT	NO_3_^−^ supplementation improves physical performance in non-athletes, particularly during long-duration open-ended tests	NA
Coggan et al. 2021 [27]	*k* = 19 Age: 17–71 years; any population	Double-blind, randomized crossover design	Total: 268 (218 male, 50 female)	PPO	Acute or chronic NO_3_^−^ intake significantly increases maximal muscle power in humans The magnitude of this effect on average (~ 5%) is likely to be of considerable practical and clinical importance	NA
D’Unienville et al. 2021 [8]	*k* = 56^a^ Age: 18–65 years; adults with various fitness levels	Randomized crossover or parallel design	Total: 956	TTE, TT, GXT	Foods rich in NO_3_^−^ provide trivial benefits for endurance exercise performance, although these effects may be food dependent Highly trained endurance athletes do not appear to benefit from consuming NO_3_^−^-rich foods	NA
Esen et al. 2023 [25]	*k* = 19 Age: > 16 years; healthy participants	Double-blind, randomized crossover design	Total: 383 (325 male, 58 female)	PPO, MPO, Time to reach PPO, MS (maximal voluntary contraction)	NO_3_^−^ supplementation may have potential to enhance PPO, MPO, and time to PPO during dynamic exercise, which may transfer to brief explosive actions commonly observed in sporting activities	NA
Evangelista et al. 2024 [24]	*k* = 27 Age: 18–45 years; apparently healthy male adults	Randomized crossover or parallel design	Total: 396	MS, ME	BRJ administration have a small ergogenic effect on ME and attenuate the decline in MS in a fatigued state in healthy male individuals	NA
Gao et al. 2021 [21]	*k* = 73 Age: > 18 years; healthy adults	Randomized or non-randomized comparative design	Total: 1061	TTE, TT, TWD, TDC, $\dot{V}$O_2_max, power output	NO_3_^−^ supplementation benefits performance-related outcomes for endurance sports	TTE: Low TT: Low TWD: Low TDC: Very low VO_2_max: Very low Power output: Low
Hogwood et al. 2023 [31]	*k* = 9 Age: 21–70 years; healthy populations and clinical populations	Single or double-blind, randomized controlled design	Total: 228 (156 male, 72 female; 158 healthy, 70 clinical populations)	TTE, $\dot{V}$ O_2_max	NO_3_^−^ supplementation combined with exercise training may not enhance exercise outcomes such as $\dot{V}$ O_2_max or TTE A trend for greater improvement in $\dot{V}$ O_2_max in healthy participants supplemented with BRJ may exist (*p* = 0.08)	TTE: High VO_2_max: Moderate
Hoon et al. 2013 [30]	*k* = 17 Age: not specified; healthy adults with no reported known disease	Randomized crossover design	Total: 184 (170 male, 14 female)	TTE, TT, GXT	NO_3_^−^ supplementation is associated with a moderate improvement in constant load TTE tasks The small positive effect on TT or GXT performance may be meaningful in an elite sport context	NA
Lago-Rodríguez et al. 2020 [23]	*k* = 5 Age: 18–80 years; healthy adults and patients with heart failure	Double-blind, randomized crossover design	Total: 60 (38 male, 22 female; 51 healthy adults, 9 patients with heart failure)	MS (isokinetic peak torque)	NO_3_^−^ supplementation does not influence isokinetic peak torque	NA
McMahon et al. 2017 [20]	*k* = 47 Age: ≥ 16 years; healthy adolescents or adults	Single or double-blind, randomized crossover design	Total: 581 (494 male, 87 female)	TTE, TT, GXT	NO_3_^−^ supplementation is likely to elicit a positive outcome for endurance exercise capacity, but less likely to be effective for TT performance	NA
Peel et al. 2021 [40]	*k* = 4^a^ Age: ≥ 18 years; healthy adults	Single or double-blind, randomized crossover or parallel design	Total: 43	Overall endurance exercise performance	NO_3_^−^ supplementation does not enhance endurance performance in the heat	NA
Senefeld et al. 2020 [41]	*k* = 80 Age: 18–42 years; young, healthy adults	Single or double-blind, randomized or counterbalanced crossover design	Total: 1335 (1179 male, 156 female)	TTE, TT, TDC	NO_3_^−^ supplementation has a clear ergogenic effect in recreationally active, young, healthy men across different exercise paradigms, but the effect size was objectively small NO_3_^−^ supplementation has more limited utility as an ergogenic aid in participants with excellent aerobic fitness that have optimized other training parameters	NA
Silva et al. 2022 [15]	*k* = 123 Age: 18–39 years; healthy adults of both sexes and any athletic status	Crossover or parallel design	Total: 1705	TTE, TT	NO_3_^−^, via beetroot juice or a high-nitrate diet, improved exercise performance, in particular, in sessions lasting between 2 and 10 min	TTE: Moderate TT: High
Silva et al. 2023 [19]	*k* = 17 Age: not specified; elite or well-trained athletes	Randomized controlled design	Total: 182 (163 male, 19 female)	TT	A significant reduction in time in against-the-clock tests after acute NO3^−^ supplementation was observed, especially for cyclists and in the form of BRJ	TT: Low
Tan et al. 2023 [22]	*k* = 6 Age: 21–29 years; resistance-trained male adults	Double-blind, randomized crossover design	Total: 92	ME (repetitions to failure), PPO, MPO	NO_3_^−^ supplementation had a small beneficial effect on some aspects of resistance exercise performance	NA
Van De Walle and Vukovich 2018 [18]	*k* = 29 Age: not specified; physically active or well-trained without any disease and injury	Crossover design	Total: 326	TT, TTE and GXT	NO_3_^−^ supplementation had a small beneficial effect on some aspects of resistance exercise performance, but there were limited studies available and the variability was large	NA
Wong et al. 2021 [28]	*k* = 17 (7 acute, 10 chronic) Age: 18–45 years; healthy active adults	Randomized crossover or parallel design	Total: 319 (257 male, 62 female)	PPO, MPO, TDC	BRJ supplementation offers no significant improvement to PPO and MPO output during HIIT or SIT	NA
Wong et al. 2022 [17]	*k* = 24 (15 acute, 8 chronic, 1 both acute and chronic) Age: 18–39 years; healthy active adults	Randomized crossover or parallel design	Total: 335 (251 male, 84 female)	TT	Chronic NO_3_^−^ supplementation improves theTT performance range from 5 to 30 min	NA

*BRJ* beetroot juice, *GRADE* Grading of Recommendations Assessment, Development and Evaluation, *GXT* graded exercise test, *HIIT* high-intensity interval training, *ME* muscular endurance, *MPO* mean power output, *MS* muscular strength, *NA* not available, *NO*_*3*_^*−*^ nitrate, *PPO* peak power output, *SIT* sprint interval training, *TDC* total distance covered, *TT* time trial, *TTE* time to exhaustion, *TWD* total work done, *VO*_*2*_*max* maximal oxygen uptake

^a^For Peel et al. [40], only studies focusing on NO3^−^ supplementation were included, while those related to other types of supplementations were excluded from the present review

### Effects of NO_3_^−^ Supplementation on Exercise Performance

**Fig. 2:**
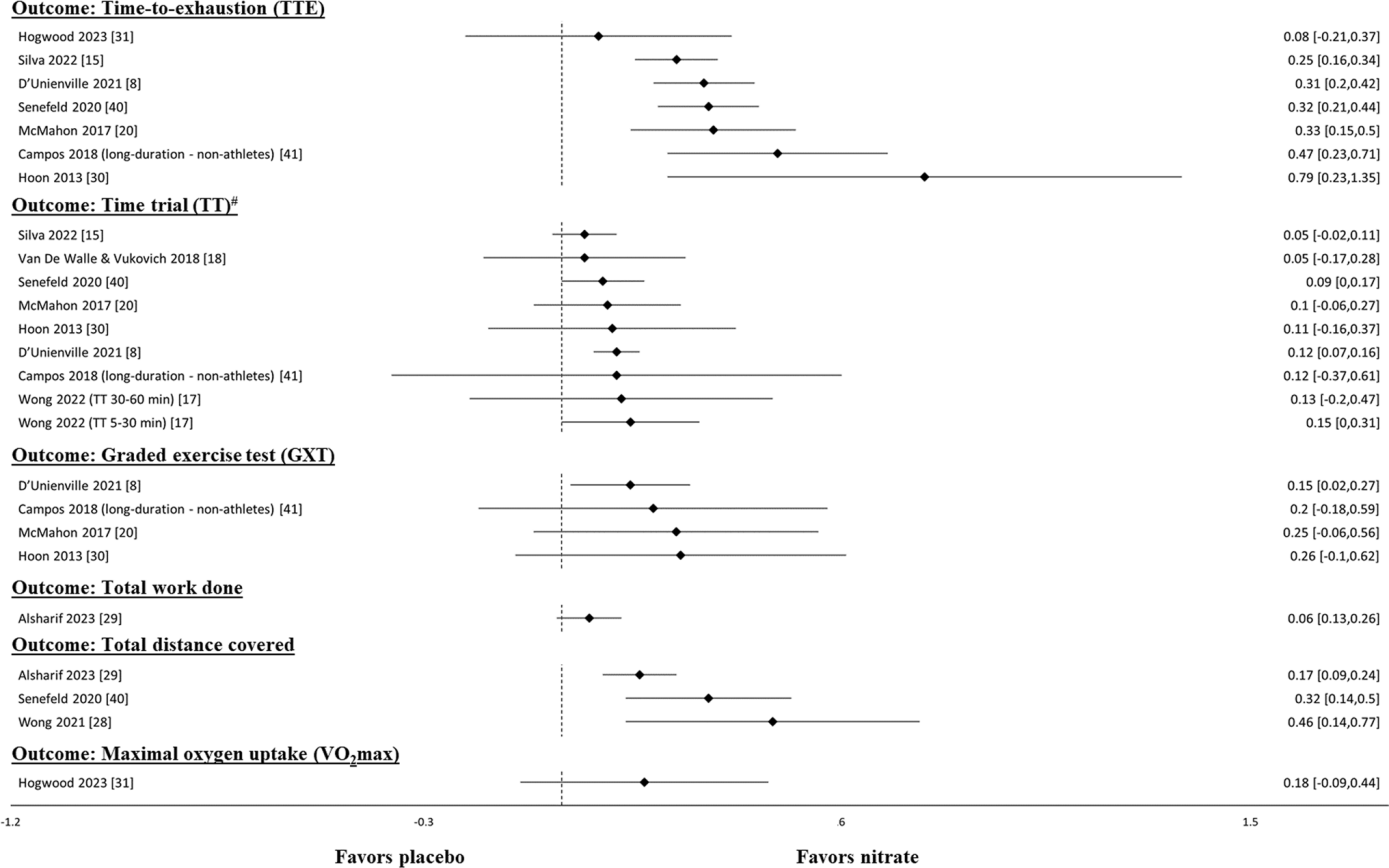
Results of meta-analyses that compared nitrate supplementation with placebo-controlled conditions for common measures of aerobic endurance performance using standardized mean differences. # a positive value indicates an improvement

The performance outcomes included in each systematic review are summarized in Table 1. Thirteen reviews involved measures related to aerobic endurance performance (Fig. 2 and Table S4 of the ESM). Seven out of eight reviews reported ergogenic effects of NO_3_^−^ supplementation on TTE, except for the study by Hogwood et al. [31], which found no significant effect (*p* = 0.58). Four analyses indicated significant improvements in total distance covered. A general trend favoring NO_3_^−^ supplementation over placebo conditions was also observed for GXT performance. However, three out of the four reviews displayed relatively wide CIs that crossed zero, indicating that the results did not reach statistical significance. Moreover, the ergogenic effect of NO_3_^−^ supplementation on TT performance was less evident with seven out of eight reviews displaying CIs that crossed zero. One meta-analysis [31], including 11 studies, reported that NO_3_^−^ supplementation did not enhance exercise training with respect to $\dot{V}$O_2_max, while another meta-analysis [29] that included seven studies reported no significant ergogenic effect of NO_3_^−^ supplementation on TWD. Gao et al. [21] included 73 studies and indicated that NO_3_^−^ supplementation benefits performance-related outcomes, including TTE and total distance traveled, for endurance sports based on an absolute mean difference.

**Fig. 3:**
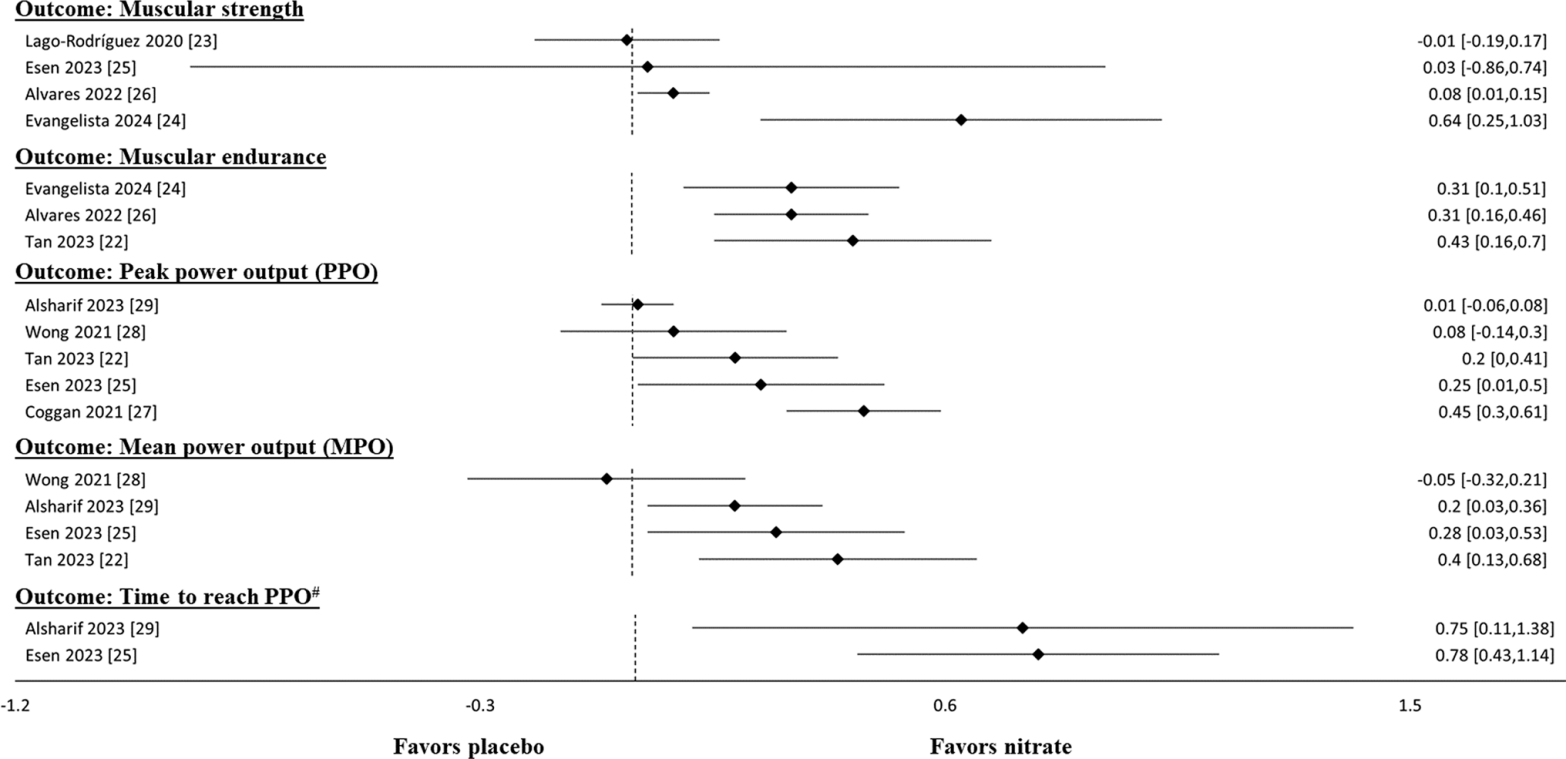
Results of meta-analyses that compared nitrate supplementation with placebo-controlled conditions for common measures of muscular fitness and power output performance using standardized mean differences

Five reviews examined measures related to muscular fitness performance (Fig. 3 and Table S5 of the ESM). All analyses indicated a significant ergogenic effect of NO_3_^−^ supplementation on muscular endurance, primarily assessed by the number of repetitions performed until failure or time of exercise until failure. Conversely, the effect of NO_3_^−^ supplementation on muscular strength, as primarily assessed by isometric maximal voluntary contraction or isokinetic peak torque, was more equivocal. Two reviews [24, 26] reported significant ergogenic effects, while another two reviews [23, 25] reported no superiority of NO_3_^−^ supplementation over placebo conditions for measures of muscular strength.

Five reviews involved measures of high-intensity power output performance (Fig. 3 and Table S5 of the ESM). The majority of these reviews (*k* = 3) reported significant improvements in terms of both PPO and MPO following NO_3_^−^ supplementation. However, two reviews reported no significant effect or less improvement with NO_3_^−^ supplementation compared to placebo conditions. Wong et al. [28] suggest that beetroot supplementation offers no significant improvement to PPO or MPO during high-intensity interval training, while Alsharif et al. [29] reported no difference between NO_3_^−^ and placebo supplementation in PPO, despite a significant improvement in MPO. Additionally, two reviews reported significant improvements in time to reach PPO [25, 29].

### Additional Meta-analyses Based on Primary Studies

To overcome the potential overlapping issues of primary studies between individual reviews (as revealed by the relatively high CCA score), additional meta-analyses were conducted using eligible primary studies included in all reviews to enhance the certainty of the findings (see Table S6 of the ESM for a tabulated summary and File S7 for forest plots). Our meta-analyses revealed that NO_3_^−^ supplementation generally had a beneficial impact on two open-ended exercise tolerance tasks, including TTE (*k* = 41; SMD: 0.33; 95% CI 0.19–0.47; *p* < 0.001] and total distance covered (*k* = 7; SMD: 0.42; 95% CI 0.09–0.76; *p* = 0.01), but not on TT tasks (*k* = 42; SMD: − 0.03; 95% CI − 0.14, 0.09; *p* = 0.65). Subgroup analyses indicated that a minimum dose of 6 mmoL/day (372 mg/day) and a chronic (> 3 days) supplementation protocol resulted in greater improvements in TTE performance. No significant effects of NO_3_^−^ supplementation were observed for GXT (*k* = 11; SMD: 0.18; 95% CI − 0.07, 0.42; *p* = 0.16), TWD (*k* = 10; SMD: 0.15; 95% CI − 0.11 to 0.40; *p* = 0.27), and $\dot{V}$ O_2_max (*k* = 34; SMD: − 0.10; 95% CI − 0.26, 0.05;* p* = 0.20).

Nitrate supplementation showed a significant ergogenic effect on muscular endurance (*k* = 22; SMD: 0.48; 95% CI 0.23–0.74; *p* < 0.001), but not muscular strength (*k* = 27; SMD: 0.05; 95% CI − 0.09, 0.19; *p* = 0.50). Subgroup analyses revealed that performance enhancements of muscular endurance were observed across all supplementation duration and dosing sub-groups. Our heterogeneity assessment revealed moderate heterogeneity (*I*^2^ = 55%; *p* = 0.001) in muscular endurance. However, this heterogeneity became non-significant (*I*^2^ = 13%; *p* = 0.29) when a primary study [46] that utilized a specific handgrip endurance test at 30% of 1 repetition maximum was removed. In addition, PPO (*k* = 27; SMD: 0.25; 95% CI 0.10–0.39; *p* < 0.001) and time to PPO (*k* = 4; SMD: − 0.76; 95% CI − 1.18, –0.33; *p* < 0.001) were improved following NO_3_^−^ supplementation, while MPO (*k* = 18; SMD: 0.14; 95% CI − 0.03, 0.32; *p* = 0.10) was not improved. Notably, sensitivity analyses using the leave-one-out method did not reveal a substantial impact of individual studies on any overall results.

### Methodological Quality of Included Reviews

Table 2 provides a summary of the AMSTAR-2 scores. Two reviews (10%) received a moderate score, while six reviews (30%) received a low score, and 12 (60%) received a critically low score (see File S8 of the ESM for scoring justifications). Specifically, only six (30%) of the reviews fully referred to a predefined methodology (item 2). None of the studies provided a list of excluded studies with reasons for exclusions (item 7), and only one study reported on the sources of funding for the included studies (item 10). Furthermore, 12 reviews (60%) did not employ appropriate methods for the statistical combination of meta-analysis results (item 11). Only 12 reviews (60%) fully used a satisfactory technique for assessing the RoB in individual studies (item 9), and seven (35%) assessed the potential impact of RoB on the results (item 12). Most reviews (*k* = 19; 95%) discussed heterogeneity in the results (item 14), and 15 (75%) investigated publication bias (item 15) when conducting meta-analyses. Results of certainty of evidence using Grading of Recommendations Assessment, Development and Evaluation (GRADE) reported by included reviews were provided in Table 1. Out of the 20 included original reviews, only three reviews provided GRADE assessments [15, 19, 21]. The reported certainty of the outcomes are as follows: TTE: “low” to “high”; TT: “low” to “high”; $\dot{V}$O_2_max: “very low” to “moderate”, TWD: “low”; total distance covered: “very low” and; power out: “low”.

**Table 2:** AMSTAR-2 ratings of systematic reviews and meta-analyses

Reference	1	2	3	4	5	6	7	8	9	10	11	12	13	14	15	16	Confidence
Alsharif et al. 2023 [29]	Y	Y	Y	PY	Y	Y	N	Y	Y	N	Y	Y	Y	Y	Y	Y	Moderate
Alvares et al. 2022 [26]	Y	N	Y	N	Y	N	N	N	Y	N	Y	N	N	N	N	Y	Critically low
Campos et al. 2018 [42]	Y	N	N	PY	N	N	N	Y	N	N	N	N	Y	Y	Y	Y	Critically low
Coggan et al. 2021 [27]	Y	Y	Y	PY	Y	Y	N	Y	N	N	Y	N	Y	Y	Y	Y	Low
D’Unienville et al. 2021 [8]	N	Y	Y	PY	Y	Y	N	PY	Y	Y	N	N	Y	Y	Y	Y	Low
Esen et al. 2023 [25]	Y	N	Y	PY	Y	Y	N	Y	PY	N	N	Y	Y	Y	N	Y	Critically low
Evangelista et al. 2024 [24]	Y	Y	Y	PY	Y	Y	N	Y	Y	N	Y	N	N	Y	N	Y	Critically low
Gao et al. 2021 [21]	Y	N	N	PY	Y	Y	N	PY	Y	N	N	Y	Y	Y	Y	Y	Critically low
Hogwood et al. 2023 [31]	N	PY	Y	PY	Y	Y	N	N	Y	N	Y	N	Y	Y	Y	Y	Moderate
Hoon et al. 2013 [30]	Y	N	Y	PY	Y	Y	N	PY	N	N	N	N	N	Y	N	N	Critically low
Lago-Rodríguez et al. 2020 [23]	Y	N	Y	PY	Y	Y	N	PY	PY	N	N	N	N	Y	Y	Y	Critically low
McMahon et al. 2017 [20]	Y	N	Y	PY	Y	Y	N	PY	PY	N	N	N	Y	Y	Y	Y	Critically low
Peel et al. 2021 [40]	Y	PY	Y	PY	Y	Y	N	PY	Y	N	Y	N	Y	Y	Y	Y	Low
Senefeld et al. 2020 [41]	Y	PY	Y	N	Y	Y	N	N	Y	N	Y	N	N	Y	Y	Y	Critically low
Silva et al. 2022 [15]	Y	PY	Y	N	Y	Y	N	N	Y	N	Y	Y	Y	Y	Y	Y	Low
Silva et al. 2023 [19]	Y	Y	Y	PY	Y	N	N	PY	Y	N	N	N	Y	Y	Y	Y	Low
Tan et al. 2023 [22]	Y	Y	Y	PY	Y	Y	N	Y	PY	N	N	Y	Y	Y	Y	Y	Low
Van De Walle and Vukovich 2018 [18]	Y	N	N	PY	N	N	N	PY	N	N	N	N	N	Y	N	Y	Critically low
Wong et al. 2021 [28]	Y	N	Y	PY	Y	Y	N	PY	Y	N	N	Y	Y	Y	Y	Y	Critically low
Wong et al. 2022 [17]	Y	N	Y	PY	Y	Y	N	Y	Y	N	N	Y	Y	Y	Y	Y	Critically low

*AMSTAR-2* A Measurement Tool to Assess Systematic Reviews Assessing the Methodological quality of SysTemAtic Review-2, *N* no, *PY* partial yes, *Y* yes

Key: Item, description:

1 Did the research questions/inclusion criteria include the components of PICO (population, intervention, comparison, outcome)?

2 Did the review contain an explicit statement that the review methods were established prior to the conduct of the review?

3 Did the review authors explain their selection of the study designs for inclusion in the review?

4 Did the review authors use a comprehensive literature search strategy?

5 Did the review authors perform study selection in duplicate?

6 Did the review authors perform data extraction in duplicate?

7 Did the review authors provide a list of excluded studies and justify the exclusions?

8 Did the review authors describe the included studies in adequate detail?

9 Did the review authors assess the risk of bias in studies that were included in the review?

10 Did the review authors report on the sources of funding for the studies included in the review?

11 If a meta-analysis was performed, did the review authors use appropriate methods for statistical combination of results?

12 If a meta-analysis was performed, did the review authors assess the potential impact of the risk of bias in individual studies on the results of the meta-analysis?

13 Did the review authors account for the risk of bias in individual studies when interpreting the results of the review?

14 Did the review authors provide a satisfactory explanation for, and discussion of, any heterogeneity observed in the results of the review?

15 If they performed quantitative synthesis, did the review authors investigate publication bias?

16 Did the review authors report any potential sources of conflict of interest, including any funding they received for conducting the review?

## Discussion

**Fig. 4:**
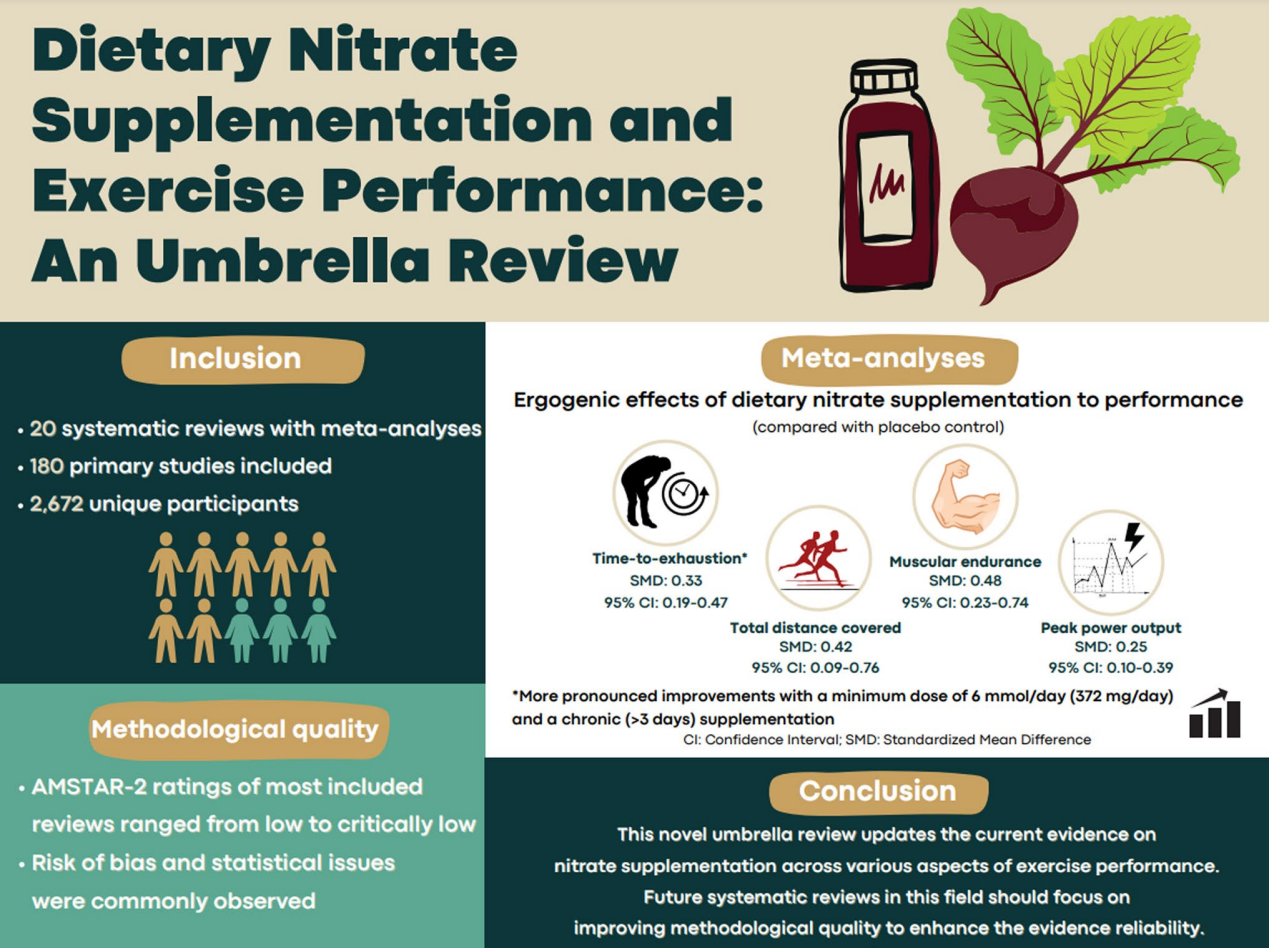
Graphical representation of the efficacy of nitrate supplementation in improving exercise performance. *AMSTAR-2* A Measurement Tool to Assess Systematic Reviews Assessing the Methodological quality of SysTemAtic Review-2, *CI* confidence interval, *SMD* standardized mean difference

The present umbrella review identified 20 systematic reviews with meta-analyses that examined the effect of NO_3_^−^ supplementation on exercise performance, involving 180 primary studies and 2672 unique participants (see Fig. 4 for the graphical representation of findings). These reviews involved a range of performance outcomes (e.g., aerobic endurance, muscular fitness, and power output tests) among diverse demographic groups, including healthy adults, resistance-trained individuals, and elite athletes. Overall, our findings revealed mixed effects of NO_3_^−^ supplementation, with some outcomes showing significant ergogenic benefits while others demonstrated non-significant effects. Additionally, we identified several methodological issues that future systematic reviews should address to enhance the reliability of the evidence.

### Methodological Issues Identified from Included Reviews

First, it is noted that out of the 20 original reviews included, only three provided explicit GRADE assessments. The certainty of the outcomes reported in these reviews was limited to six performance measures (e.g., TTE, TT, $\dot{V}$O_2_max, TWD, total distance covered, and power output) and showed a diverse range of judgments, varying from “very low” to “high”. These observations suggest a lack of robust evaluation across individual reviews, which may contribute to the equivocal effects reported on NO_3_^−^ supplementation. Second, a relatively high proportion of included systematic reviews were rated as low (*k* = 6) or critically low (*k* = 12) in quality based on the AMSTAR-2 rating, and did not strictly adhere to the Preferred Reporting Items for Systematic Reviews and Meta-Analysis (PRISMA) guidelines, which currently present a widely accepted standard for reporting a meta-analysis. For instance, Senefeld et al. [41] and Van De Walle et al. [18] did not report the RoB of each included study, which could have undermined the confidence in the validity and reliability of the review’s findings. Furthermore, two reviews [19, 21] used absolute units (mean difference) instead of the SMD to synthesize meta-analysis results. Because of the ease of interpretation, reporting mean differences is preferred when summarizing a body of literature that quantifies a singular outcome variable with consistent units of measure while using extremely comparable testing protocols [38]. However, absolute units can be problematic given the heterogeneity in exercise protocol design in the NO_3_^−^ literature, even for the same general type of performance outcome (e.g., endurance tests with various pre-determined distances or intensities). Future NO_3_^−^ reviews should favor SMDs over absolute units to enable more meaningful comparisons across studies, as the preferred approach based on existing guidelines [38].

Moreover, only a small proportion of reviews fully referred to a predefined methodology (i.e., adherence to a written protocol with independent verification by a registry or another independent body). None of the reviews provided a list of excluded studies with reasons for exclusions and only one review reported on the sources of funding for the included studies, which may potentially indicate publication bias. Furthermore, 12 reviews did not employ appropriate methods for the statistical combination of meta-analysis results. In particular, ten reviews used multiple data points from individual studies without accounting for the likely dependence between those points [8, 17, 19–23, 25, 28, 42], while two applied a fixed-effect meta-analytic model [18, 30], which is unrealistic given that it is unlikely NO_3_^−^ supplementation has a single true effect across samples. There were also six reviews utilized RoB/quality assessments tools (i.e., either the PEDro tool [20, 22, 23, 25] or “customized” tools [27, 42]) that do not fully assess bias arising from the selection of reported outcomes, while 12 reviews did not perform analyses to investigate the possible impact of RoB on summary estimates. Taken together, these observations underscore the importance of exercising caution when interpreting certain included reviews and highlights the need for well-conducted systematic reviews in this field.

### Effects of NO_3_^−^ Supplementation on Aerobic Endurance Performance

Despite the potential methodological issues identified in the included reviews, we conducted a re-analysis of the data through large-scale meta-analyses using the 180 eligible primary studies from all reviews. This approach can help address potential overlaps among primary studies and clarify the results from previous reviews. Our results indicated equivocal effects regarding the effects of NO_3_^−^ supplementation on various parameters of aerobic endurance performance. Notably, NO_3_^−^ supplementation improved TTE and total distance covered, both of which are open-ended exercise tolerance tasks. The potential mechanisms underlying such ergogenic effects have been discussed and outlined in detail elsewhere [2, 47]. Briefly, NO_3_^−^ supplementation has been shown to increase the bioavailability of NO, which can lead to improved muscle oxygenation, mitochondrial efficiency, and enhanced contractile function [2, 47]. These physiological adaptations may collectively contribute to improved endurance capacity.

Intriguingly, in contrast to the open-ended exercise tolerance tasks, exercise tests that assessed the time taken to complete a fixed distance or work (i.e., TT tests) showed more equivocal results among the included reviews [8, 15, 17, 18, 20, 30, 41, 42], and our meta-analysis of all included primary studies did not reveal significant improvements. This distinction is important, as open-ended exercise tasks involving exercising until exhaustion have been suggested to have a greater variability, partly owing to psychological factors such as boredom and motivation [48, 49]. In contrast, the negligible effect on TT performance may be due to the complex interplay of physiological and psychological factors that influence pacing and performance during self-paced exercise [48, 49]. Early work on the reliability of physical performance tests [50] has suggested that an ~ 15% change in TTE in a constant-power test is equivalent to a 1% change in power output in a TT test. It might also be possible that these differences are so small that they are often undetectable in research settings because of typical biological or equipment variability [51]. Furthermore, our meta-analysis findings showed no significant improvements in GXT and $\dot{V}$O_2_max following NO_3_^−^ supplementation. One possible explanation is that the ergogenic effects of NO_3_^−^ may be more pronounced in endurance activities that require sustained effort rather than in short bursts of maximal effort [21]. The benefits of increased NO production from NO_3_^−^ are more relevant during prolonged exercise, where oxygen delivery and utilization are critical, rather than shorter duration, high-intensity aerobic efforts that characterize most GXT or $\dot{V}$O_2_max testing.

### Effects of NO_3_^−^ Supplementation on Muscular Fitness Performance

Our review also found a mixed effect of NO_3_^−^ supplementation on muscular fitness performance. Notably, positive effects of NO_3_^−^ supplementation on measures of muscular endurance were observed. These improvements are likely mediated by the reduced ATP cost of force production and spared muscle phosphocreatine stores during submaximal contraction, as well as enhanced muscle blood flow, oxygen delivery, mitochondrial respiration, and calcium handling, as previously highlighted [6, 52, 53]. However, the effects of NO_3_^−^ supplementation on muscular strength were more ambiguous among individual systematic reviews [23–25, 54], and our meta-analysis based on all included primary studies did not reveal significant improvements. This may be because the vascular effects of NO_3_^−^, such as enhanced vasodilation and blood flow, have a greater impact on sustaining aerobic metabolism during endurance tasks compared to their influence on maximal force production. Furthermore, it is possible that strength-oriented exercise tests tend to involve muscular contraction speeds that are too slow to maximally benefit from the effects of NO_3_^−^ on muscle contractile properties. As reviewed by Coggan and Peterson [55], multiple studies have shown NO_3_^−^ supplementation to increase force production at higher velocities of contraction, but not at lower velocities. Nevertheless, the exact reasons behind the seemingly divergent effects on muscular strength versus endurance remain speculative, and it is acknowledged that measuring the energetic cost of activation in skeletal muscle can be challenging [52]. Additional research is needed to fully elucidate these potential mechanistic differences.

### Effects of NO_3_^−^ Supplementation on High-Intensity Power Output Performance

Our overall meta-analysis revealed that NO_3_^−^ supplementation can improve various parameters of high-intensity power output performance, including PPO and time to reach PPO. These benefits may be mediated by NO_3_^−^-derived NO that enhances the rate of phosphocreatine resynthesis and the capacity of the anaerobic glycolytic pathway [7]. The improvements in high-intensity power output with NO_3_^−^ supplementation may be particularly relevant for sports and activities that require rapid explosive movements, such as sprinting, jumping, and plyometric training, as the increased power output could potentially translate to enhanced performance in these events [25, 27]. Furthermore, it has been suggested that NO_3_^−^ supplementation may have a stronger effect on initial force production of type II muscle fibers [28]. This may explain the improvement in time to reach PPO observed here, but not in MPO which encompasses a broader range of intensities and duration, where the advantages of NO_3_^−^ were not observed to translate effectively.

### Potential Moderators of NO_3_^−^ Supplementation Effects

To examine the potential moderators of NO_3_^−^ supplementation effects, we conducted subgroup analyses based on two key components of the supplementation protocol — duration and daily intake dose. Our findings indicated that a minimum dose of 6 mmoL/day (372 mg/day) and a chronic (> 3 days) supplementation protocol resulted in greater improvements in TTE performance. These findings are generally consistent with the subgroup analysis or linear meta-regression analysis performed by the individual reviews, which suggested that chronic (i.e., multiple-day) supplementation of NO_3_^−^ confers greater benefits in improving performance compared with acute supplementation [17, 29, 30, 41], and a minimum of 5 mmoL/day (310 mg/day) would be recommended for performance benefits [15, 18, 41]. Nonetheless, the ergogenic effects of muscular endurance were observed across all supplementation duration and dosing sub-groups. These findings imply that while higher doses and longer durations may yield greater benefits, there remains potential for performance improvement across various supplementation strategies, depending on the specific training and performance contexts.

Aside from the exercise type, duration and dosing factors, several individual reviews [8, 30, 41, 42] indicated that the ergogenic benefits of NO_3_^−^ supplementation appear to be more pronounced in non-athletes and recreationally active individuals, as compared with highly trained athletes. The underlying mechanisms likely involve a “ceiling effect,” where highly fit individuals may have already optimized their physiological systems for performance and have less room for improvement in terms of oxygen delivery, mitochondrial function, and NO bioavailability, compared with their less-fit counterparts [56]. It is also suggested that well-trained individuals tend to have higher baseline levels of NO_3_^−^ than untrained individuals as training can enhance the production of NO via the NO synthase pathway [57]. Another suggestion is that highly trained or elite athletes have a greater habitual NO_3_^−^ intake through their diet because of higher overall energy intakes, but this suggestion has been challenged [58] as the overall doses are lower than those typically obtained by supplemental NO_3_^−^ doses.

Additionally, several reviews reported that other supplementation and exercise components may moderate the effects of NO_3_^−^ on exercise performance. For instance, NO_3_^−^ ingestion appears to be more effective when exercise is performed under hypoxic conditions [15] or in a fatigued state [24]. Furthermore, hygiene practices that negatively impact oral microbiota may diminish the ergogenic effects of NO_3_^−^, while beetroot juice and a high-NO_3_^−^ diet offer greater benefits than NO_3_^−^ salts [15]. However, other reviews either did not observe clear moderation effects or conduct specific analyses on these parameters, likely owing to significant heterogeneity in study design or insufficient data. Future studies should continue to explore the impact of various moderators on exercise performance, by employing additional high-quality randomized controlled trials and a moderation analysis.

### Strengths and Limitations

The strengths of this umbrella review include adherence to PRIOR guidelines and the use of widely recognized benchmarks (e.g., AMSTAR-2) to assess the scientific rigor of the included systematic reviews. We focused exclusively on the highest level of evidence (i.e., systematic reviews with meta-analyses) to ensure the robustness of our analyses. Our additional meta-analyses based on primary studies further enhanced the accuracy and consistency of our summarized results. Moreover, sensitivity analyses using the leave-one-out method showed that no individual study had a substantial impact on the overall results, reinforcing the robustness of our findings. However, several limitations were acknowledged. A notable limitation of existing research is the predominant focus on men, which may have overlooked potential sex differences in responses to NO_3_^−^ supplementation. In the 11 reviews that reported on the sex ratio of the included participants, only 7–37% of the pooled sample participants were women, while two reviews solely involved male participants [22, 24]. Only three reviews have conducted separate analyses on men and women [27, 31, 41]. It has been suggested that differences in sex hormone composition and the associated disparity in muscle mass between men and women may impact the storage, utilization, and retention of NO_3_^−^ within the body following supplementation [59]. Additionally, there is a lack of studies focusing on youth and older populations, who have distinct physiological characteristics, such as variations in muscle mass, hormonal profiles, and cardiovascular function, which can influence their adaptation to NO_3_^−^ supplementation [60, 61]. Therefore, it is crucial for future studies and systematic reviews to specifically examine the effects of NO_3_^−^ on exercise performance across both sex and various age groups. Finally, authors of future NO_3_^−^ studies should be sure to include detailed information about the source of NO_3_^−^ utilized and, if possible, the results of independent testing to verify the NO_3_^−^ content of the product [62]. This information is critically important, as commercially available beetroot juice products display considerable within-product and between-product variation in measured NO_3_^−^ content, with large differences between labeled values and measured values often observed [63]. The same is true for studies that utilize whole-food sources of NO_3_^−^, as the naturally occurring NO_3_^−^ content of both conventional and organic vegetables can also vary considerably based on regional differences, soil quality, growing conditions, and storage conditions [64].

## Conclusions

This novel umbrella review provides comprehensive and up-to-date evidence on the effects of NO_3_^−^ supplementation across various exercise performance outcomes. Our findings indicate that NO_3_^−^ supplementation improves performance in TTE tasks, total distance covered, muscular endurance, PPO, and time to PPO, but does not show ergogenic effects on other performance outcomes. Future systematic reviews in this field should focus on improving methodological quality in reporting to enhance the reliability of the evidence.

## Supplementary Information

Below is the link to the electronic supplementary material.

Supplementary file1 (DOCX 15 KB)

Supplementary file2 (DOCX 22 KB)

Supplementary file3 (DOCX 36 KB)

Supplementary file4 (DOCX 20 KB)

Supplementary file5 (DOCX 19 KB)

Supplementary file6 (DOCX 34 KB)

Supplementary file7 (DOCX 14426 KB)

Supplementary file8 (PDF 826 KB)

## Declarations

### Availability of Data and Material

The datasets analyzed in this review are available from the corresponding author on reasonable request.
