# Supplementary material for: Dietary Nitrate Supplementation and Exercise Performance: An Umbrella Review of 20 Published Systematic Reviews with Meta-analyses
Source: Sports Med. 2025 Mar 14;55(5):1213–31. doi: 10.1007/s40279-025-02194-6 (PMC12106159; doi:10.1007/s40279-025-02194-6)
Supplement: Supplementary file 1 — Supplementary file1 (DOCX 15 KB) [file 40279_2025_2194_MOESM1_ESM.docx]

**Supplementary Table S1 Search Strategy**

**For Medline**

| **Terms** | **Search Strategy** |
| --- | --- |
| Dietary nitrate | (“nitrate”[tw] OR “beetroot”[tw] OR “nitrite” [tw] OR “nitric oxide”[tw]) |
| Exercise performance | ("exercise" [tw] OR “sport” [tw]OR "training" [tw] OR "muscle" [tw] OR "physical performance"[tw]) |
| Reviews | (“systematic review”[tw] OR “meta analysis”[tw]) |

**For EMBASE, Cochrane Database, CINAHL, Scopus, SPORTDiscus, and Web of Science**

| **Terms** | **Search Strategy** |
| --- | --- |
| Dietary nitrate | (“nitrate” OR “beetroot” OR “nitrite” OR “nitric oxide”) |
| Exercise performance | ("exercise" OR “sport” OR "training" OR "muscle" OR "physical performance") |
| Reviews | (“systematic review” OR “systematic literature review” OR “systematic” OR “review” OR “cochrane database syst rev” OR “meta-analysis” OR “metaanalysis” OR “meta analysis” OR “meta-analytic review” OR “meta analy*” OR “meta-analy*” OR “metaanaly*” OR “metareview” OR “meta-review”) |
