# Supplementary material for: Dietary Nitrate Supplementation and Exercise Performance: An Umbrella Review of 20 Published Systematic Reviews with Meta-analyses
Source: Sports Med. 2025 Mar 14;55(5):1213–31. doi: 10.1007/s40279-025-02194-6 (PMC12106159; doi:10.1007/s40279-025-02194-6)
Supplement: Supplementary file 2 — Supplementary file2 (DOCX 22 KB) [file 40279_2025_2194_MOESM2_ESM.docx]

**Supplementary Table S2** List of excluded studies

| **Reference** | **Journal** | **Title** | **Reason** |
| --- | --- | --- | --- |
| Abreu et al 2023 | Journal of the International Society of Sports Nutrition | Effects of dietary supplements on athletic performance in elite soccer players: a systematic review | no meta-analysis |
| Afrisham et al 2023 | British Journal of Nutrition | The effects of beetroot and nitrate supplementation on body composition: a GRADE-assessed systematic review and meta-analysis | not performance-related outcomes |
| Alshafie et al 2021 | Clinical Nutrition Espen | Efficacy of dietary nitrate-rich beetroot juice supplementation in patients with chronic obstructive pulmonary disease (COPD): A systematic review and meta-analysis | not performance-related outcomes |
| Alsulayyim et al 2021 | Bmj Open Respiratory Research | Impact of dietary nitrate supplementation on exercise capacity and cardiovascular parameters in chronic respiratory disease: a systematic review and meta-analysis | focus on clinical populations |
| Alvarez et al 2021 | Retos-Nuevas Tendencias En Educacion Fisica Deporte Y Recreacion | Effect of beet juice supplementation (BJ) on neuromuscular response: a systematic review | not an English article |
| Amirpoor et al 2022 | Current Problems in Cardiology | Effect of Beetroot Consumption on Serum Lipid Profile: A Systematic Review and Meta-Analysis | not performance-related outcome |
| Anderson et al 2022 | Journal of Strength and Conditioning Research | Effects of Nitrate Supplementation on Muscle Strength and Mass: A Systematic Review | no meta-analysis |
| Arefirad et al 2022 | Frontiers in Physiology | Effect of exercise training on nitric oxide and nitrate/nitrite (NOx) production: A systematic review and meta-analysis | not dietary nitrate supplementation |
| Ashor et al 2017 | Journal of Hypertension | Medium-term effects of dietary nitrate supplementation on systolic and diastolic blood pressure in adults: a systematic review and meta-analysis | not performance-related outcomes |
| Ashworth et al 2015 | Public Health Nutrition | High-nitrate vegetable diet increases plasma nitrate and nitrite concentrations and reduces blood pressure in healthy women | not performance-related outcomes |
| Bahadoran et al 2015 | Nitric Oxide-Biology and Chemistry | Is dietary nitrate/nitrite exposure a risk factor for development of thyroid abnormality? A systematic review and meta-analysis | not performance-related outcomes |
| Barnard et al 2022 | Nutrients | The Impact of Dietary Factors on the Sleep of Athletically Trained Populations: A Systematic Review | no meta-analysis |
| Beijers et al 2018 | Clinical Nutrition | The effect of acute and 7-days dietary nitrate on mechanical efficiency, exercise performance and cardiac biomarkers in patients with chronic obstructive pulmonary disease | not performance-related outcomes |
| Benjamim et al 2024 | Free Radical Biology and Medicine | Effects of dietary inorganic nitrate on blood pressure during and post-exercise recovery: A systematic review and meta-analysis of randomized placebo-controlled trials | not performance-related outcomes |
| Beresewicz & Gajos-Draus 2016 | Kardiologia Polska | Enjoy your heart-beets. The role of dietary inorganic nitrate in cardiovascular health | not a systematic review |
| Blake et al 2021 | Journal of Science and Medicine in Sport | Polyphenol consumption and endurance exercise performance: A systematic review and meta-analysis of randomised controlled trials | conference abstract only |
| Calvo et al 2020 | Nutrients | Influence of Nitrate Supplementation on Endurance Cyclic Sports Performance: A Systematic Review | no meta-analysis |
| Clifford et al 2019 | Critical Reviews in Food Science and Nutrition | Effects of inorganic nitrate and nitrite consumption on cognitive function and cerebral blood flow: A systematic review and meta-analysis of randomized clinical trials | not performance-related outcomes |
| Delleli et al 2023 | Nutrients | Does Beetroot Supplementation Improve Performance in Combat Sports Athletes? A Systematic Review of Randomized Controlled Trials | no meta-analysis |
| Dominguez et al 2017 | Nutrients | Effects of Beetroot Juice Supplementation on Cardiorespiratory Endurance in Athletes. A Systematic Review | no meta-analysis |
| Ferrada-Contreras et al 2023 | Nutrients | Does Co-Supplementation with Beetroot Juice and Other Nutritional Supplements Positively Impact Sports Performance?: A Systematic Review | no meta-analysis |
| Flood & Fleisher 2007 | American Family Physician | Preparation of the cardiac patient for noncardiac surgery | not dietary nitrate supplementation |
| Fontes et al 2019 | Frontiers in Sustainable Food Systems | Combining Environmental Monitoring and Remote Sensing Technologies to Evaluate Cropping System Nitrogen Dynamics at the Field-Scale | not dietary nitrate supplementation |
| Gamonales et al 2022 | International Journal of Environmental Research and Public Health | Effectiveness of Nitrate Intake on Recovery from Exercise-Related Fatigue: A Systematic Review | no meta-analysis |
| Gonzalez & Trexler 2020 | Journal of Strength & Conditioning Research | Effects of Citrulline Supplementation on Exercise Performance in Humans: A Review of the Current Literature | not a systematic review |
| Guerra et al 2022 | Rbne-Revista Brasileira De Nutricao Esportiva | Nitrate derived from beet juice and its influences on high-intensity exercise: a systematic review of randomized clinical trials | no meta-analysis |
| Hlinsky et al 2020 | Nutrients | Effects of Dietary Nitrates on Time Trial Performance in Athletes with Different Training Status: Systematic Review | no meta-analysis |
| Hopkins et al 2016 |  | The Triumph of Technology for Athletes at the 21st Annual Meeting of the European College of Sport Science | conference abstract only |
| Jones et al 2022 | Journal of Dietary Supplements | The Effect of Nitrate-Rich Beetroot Juice on Markers of Exercise-Induced Muscle Damage: A Systematic Review and Meta-Analysis of Human Intervention Trials | not performace-related outcomes |
| Kaufman et al 2022 | Current Sports Medicine Reports | The Impact of Supplements on Sports Performance for the Trained Athlete: A Critical Analysis | not a systematic review |
| Kerley 2017 | Current Opinion in Clinical Nutrition & Metabolic Care | Dietary nitrate as modulator of physical performance and cardiovascular health | not a systematic review |
| Kim & Kim 2020 | Nutrients | Nutritional Strategies to Optimize Performance and Recovery in Rowing Athletes | no meta-analysis |
| Klemsdal & Gjesdal 1992 | Cardiovascular Drugs and Therapy | The effect of transdermal nitroglycerin on exercise tolerance in relation to patch application time-a meta-analysis | not dietary nitrate supplementation |
| Lara et al 2015 | Maturitas | Effects of handgrip exercise or inorganic nitrate supplementation on 24-h ambulatory blood pressure and peripheral arterial function in overweight and obese middle age and older adults: A pilot RCT | not a systematic review |
| Linoby et al 2020 |  | The Role of Fitness Status in the Performance-Enhancing Effects of Dietary Inorganic Nitrate Supplementation: Meta-analysis and Meta-regression Analysis | only a book section |
| Long et al 2020 | Journal of International Medical Research | Effect of nitrate treatment on functional capacity and exercise time in patients with heart failure: a systematic review and meta-analysis | focus on clinical populations |
| Lorenzo Calvo et al 2020 | Nutrients | Influence of Nitrate Supplementation on Endurance Cyclic Sports Performance: A Systematic Review | no meta-analysis |
| Lowings et al 2017 | International Journal of Sport Nutrition and Exercise Metabolism | Effect of Dietary Nitrate Supplementation on Swimming Performance in Trained Swimmers | not a systematic review |
| Macuh & Knap 2021 | Nutrients | Effects of nitrate supplementation on exercise performance in humans: A narrative review | not a systematic review |
| Martin-Olmedo et al 2023 | Journal of Strength and Conditioning Research | Manuscript Clarification for "Effects of Nitrate Supplementation on Muscle Strength and Mass: A Systematic Review" | commentary only |
| Meirelles & Spanolonse 2023 | Rbne-Revista Brasileira De Nutricao Esportiva | Effects of nitrate supplementation on running performance: a systematic review | no meta-analysis |
| Nyawose et al 2022 | Beverages | The Effects of Consuming Amino Acids L-Arginine, L-Citrulline (and Their Combination) as a Beverage or Powder, on Athletic and Physical Performance: A Systematic Review | no meta-analysis |
| Park et al 2021 | Medicine and Science in Sports and Exercise | The Effect Of Beetroot Juice Supplementation On Muscle Fatigue In Healthy Adults; A Meta-analysis | conference abstract only |
| Pawlak-Chaouch et al 2016 | Nitric Oxide-Biology and Chemistry | Effect of dietary nitrate supplementation on metabolic rate during rest and exercise in human: A systematic review and a meta-analysis | not performance-related outcomes |
| Quaresma et al 2021 | Nutrition | Effects of diet interventions, dietary supplements, and performance-enhancing substances on the performance of CrossFit-trained individuals: A systematic review of clinical studies | no meta-analysis |
| Ramos Álvarez et al 2021 | Retos | Effect of beet juice supplementation (BJ) on neuromuscular response: A systematic review | not an English article |
| Renji et al 2023 | Age and Ageing | The Effects of Dietary Nitrate Supplementation on Physical Performance in Older People - a Systematic Review | no meta-analysis |
| Rojano-Ortega et al 2022 | Sports & Health | Effects of Beetroot Supplementation on Recovery After Exercise-Induced Muscle Damage: A Systematic Review | no meta-analysis |
| Rojas-Valverde et al 2021 | Critical Reviews in Food Science and Nutrition | Effectiveness of beetroot juice derived nitrates supplementation on fatigue resistance during repeated-sprints: a systematic review | no meta-analysis |
| Saleh et al 2021 | Journal of Health and Translational Medicine | Nitrate supplementation improved aerobic endurance performance: An umbrella review | conference abstract only |
| San Juan et al 2020 | Nutrients | Effects of Dietary Nitrate Supplementation on Weightlifting Exercise Performance in Healthy Adults: A Systematic Review | no meta-analysis |
| Smith et al 2022 | Nutrients | Auditing the Representation of Female Versus Male Athletes in Sports Science and Sports Medicine Research: Evidence-Based Performance Supplements | not a systematic review |
| Stanaway et al 2017 | Nutrients | Performance and Health Benefits of Dietary Nitrate Supplementation in Older Adults: A Systematic Review | no meta-analysis |
| Tan et al 2022 | International Journal of Environmental Research & Public Health | The Effects of Dietary Nitrate Supplementation on Explosive Exercise Performance: A Systematic Review | no meta-analysis |
| Trexler et al 2019 | Sports Medicine | Acute Effects of Citrulline Supplementation on High-Intensity Strength and Power Performance: A Systematic Review and Meta-Analysis | not dietary nitrate supplementation |
| Vicente-Salar et al 2020 | Nutrients | Nutritional Ergogenic Aids in Racquet Sports: A Systematic Review | no meta-analysis |
| Vicente-Salar et al 2022 | Nutrients | Nutritional Ergogenic Aids in Combat Sports: A Systematic Review and Meta-Analysis | not dietary nitrate supplementation |
| Viribay et al 2020 | Nutrients | Effects of Arginine Supplementation on Athletic Performance Based on Energy Metabolism: A Systematic Review and Meta-Analysis | not dietary nitrate supplementation |
| Viribay et al 2022 | Nutrients | Effects of Citrulline Supplementation on Different Aerobic Exercise Performance Outcomes: A Systematic Review and Meta-Analysis | not dietary nitrate supplementation |
| Vitale & Getzin 2019 | Nutrients | Nutrition and supplement update for the endurance athlete: Review and recommendations | not a systematic review |
| Wang et al 2024 | Respiratory Medicine | Dietary nitrate supplementation to enhance exercise capacity in patients with COPD: Evidence from a meta-analysis of randomized controlled trials and a network pharmacological analysis | focus on clinical populations |
| Zamani et al 2021 | Critical Reviews in Food Science and Nutrition | The benefits and risks of beetroot juice consumption: a systematic review | no meta-analysis |
