# Supplementary material for: Dietary Nitrate Supplementation and Exercise Performance: An Umbrella Review of 20 Published Systematic Reviews with Meta-analyses
Source: Sports Med. 2025 Mar 14;55(5):1213–31. doi: 10.1007/s40279-025-02194-6 (PMC12106159; doi:10.1007/s40279-025-02194-6)
Supplement: Supplementary file 3 — Supplementary file3 (DOCX 36 KB) [file 40279_2025_2194_MOESM3_ESM.docx]

**Supplementary Table S3** List of included primary studies

| 1 | Amano 2018 | Influence of dietary nitrate supplementation on local sweating and cutaneous vascular responses during exercise in a hot environment | 8 |
| --- | --- | --- | --- |
| 2 | Arnold 2015 | Beetroot juice does not enhance altitude running performance in well-trained athletes | 10 |
| 3 | Aucouturier 2015 | Effect of dietary nitrate supplementation on tolerance to supramaximal intensity intermittent exercise | 12 |
| 4 | Bailey 2009 | Dietary nitrate supplementation reduces the O2 cost of low-intensity exercise and enhances tolerance to high-intensity exercise in humans | 8 |
| 5 | Bailey 2010 | Dietary nitrate supplementation enhances muscle contractile efficiency during knee-extensor exercise in humans | 7 |
| 6 | Bailey 2015 | Inorganic nitrate supplementation improves muscle oxygenation, O2 uptake kinetics and exercise tolerance at high but not low pedal rates | 7 |
| 7 | Balsalobre-Fernández 2018 | The effects of beetroot juice supplementation on exercise economy, rating of perceived exertion and running mechanics in elite distance runners: A double-blinded, randomized study | 12 |
| 8 | Bender 2018 | Acute beetroot juice administration improves peak isometric force production in adolescent males | 12 |
| 9 | Bernardi 2018 | Acute Supplementation with Beetroot Juice Does Not Enhance Exercise Performance among Well-trained Athletes: A Randomized Crossover Study | 10 |
| 10 | Berry 2020 | A randomized controlled trial of nitrate supplementation in well-trained middle and older-aged adults | 15 |
| 11 | Bescos 2011 | Acute administration of inorganic nitrate reduces VO(2peak) in endurance athletes | 11 |
| 12 | Bescos 2012 | Sodium nitrate supplementation does not enhance performance of endurance athletes | 13 |
| 13 | Betteridge 2015 | No effect of acute beetroot juice ingestion on oxygen consumption, glucose kinetics or skeletal muscle metabolism during submaximal exercise in males | 8 |
| 14 | Bond 2012 | Dietary nitrate supplementation improves rowing performance in well-trained rowers | 14 |
| 15 | Boorsma 2014 | Beetroot juice supplementation does not improve performance of elite 1500-m runners | 8 |
| 16 | Bourdillon 2015 | Effect of oral nitrate supplementation on pulmonary hemodynamics during exercise and time trial performance in normoxia and hypoxia: a randomized controlled trial | 12 |
| 17 | Breese 2013 | Beetroot juice supplementation speeds O2 uptake kinetics and improves exercise tolerance during severe-intensity exercise initiated from an elevated metabolic rate | 9 |
| 18 | Breese 2017 | The effect of dietary nitrate supplementation on the spatial heterogeneity of quadriceps deoxygenation during heavy‐intensity cycling | 8 |
| 19 | Buck 2015 | Effects of sodium phosphate and beetroot juice supplementation on repeated-sprint ability in females | 13 |
| 20 | Callahan 2017 | Single and combined effects of beetroot crystals and sodium bicarbonate on 4-km cycling time trial performance | 8 |
| 21 | Carpentier et al., 2015 | Nitrate supplementation, exercise, and kidney function: are there detrimental effects? | 13 |
| 22 | Casado 2021 | Influence of Sex and Acute Beetroot Juice Supplementation on 2 KM Running Performance | 24 |
| 23 | Cermak 2012(a) | No improvement in endurance performance after a single dose of beetroot juice | 20 |
| 24 | Cermak 2012(b) | Nitrate supplementation’s improvement of 10-km time-trial performance in trained cyclists | 12 |
| 25 | Christensen 2013 | Influence of nitrate supplementation on VO(2) kinetics and endurance of elite cyclists | 10 |
| 26 | Christensen 2017 | Effects of nitrate supplementation in trained and untrained muscle are modest with initial high plasma nitrite levels | 17 |
| 27 | Clifford 2016 | Effects of beetroot juice on recovery of muscle function and performance between bouts of repeated sprint exercise. | 20 |
| 28 | Clifford et al., 2016 | The effects of beetroot juice supplementation on indices of muscle damage following eccentric exercise | 30 |
| 29 | Clifford et al., 2017 | Beetroot juice is more beneficial than sodium nitrate for attenuating muscle pain after strenuous eccentric-bias exercise | 30 |
| 30 | Cocksedge 2020 | Influence of muscle oxygenation and nitrate-rich beetroot juice supplementation on O2 uptake kinetics and exercise tolerance | 10 |
| 31 | Coggan 2015(a) | Effect of acute dietary nitrate intake on maximal knee extensor speed and power in healthy men and women | 12 |
| 32 | Coggan 2020 | A Single Dose of Dietary Nitrate Increases Maximal Knee Extensor Angular Velocity and Power in Healthy Older Men and Women. | 12 |
| 33 | Coggan et al.,2018 | Dietary nitrate-induced increases in human muscle power: high versus low responders | 20 |
| 34 | Collins & Kearns, 2020 | The effect of beetroot supplementation on high-intensity functional training performance | 24 |
| 35 | Corry 2015 | Dietary nitrate enhances power output during the early phases of maximal intensity sprint cycling | 10 |
| 36 | Craig 2018 | Effect of dietary nitrate supplementation on conduit artery blood flow, muscle oxygenation, and metabolic rate during handgrip exercise | 9 |
| 37 | Crum 2017 | The effect of acute pomegranate extract supplementation on oxygen uptake in highly-trained cyclists during high-intensity exercise in a high altitude environment | 8 |
| 38 | Crum 2018 | Multiday Pomegranate Extract Supplementation Decreases Oxygen Uptake During Submaximal Cycling Exercise, but Cosupplementation With N-acetylcysteine Negates the Effect | 8 |
| 39 | Cuenca 2018 | Effects of beetroot juice supplementation on performance and fatigue in a 30-s all-out sprint exercise: a randomized, double-blind cross-over study | 15 |
| 40 | Daab et al., 2021 | Chronic beetroot juice supplementation accelerates recovery kinetics following simulated match play in soccer players | 13 |
| 41 | de Castro 2018 | Beetroot juice supplementation does not modify the 3-km running performance in untrained women | 8 |
| 42 | de Castro 2019 | Effect of beetroot juice supplementation on 10-km performance in recreational runners | 14 |
| 43 | de Castro 2019 (a) | Effects of chronic beetroot juice supplementation on maximum oxygen uptake, velocity associated with maximum oxygen uptake, and peak velocity in recreational runners: a double-blinded, randomized and crossover study | 13 |
| 44 | de Oliveira 2017 | Acute effect of dietary nitrate on forearm muscle oxygenation, blood volume and strength in older adults: A randomized clinical trial. | 12 |
| 45 | de Oliveira 2018 | Beetroot-based gel supplementation improves handgrip strength and forearm muscle O2 saturation but not exercise tolerance and blood volume in jiu-jitsu athletes | 12 |
| 46 | de Oliveira 2020 | A single oral dose of beetroot-based gel does not improve muscle oxygenation parameters, but speeds up handgrip isometric strength recovery in recreational combat sports athletes. | 14 |
| 47 | De Smet et al., 2016 | Nitrate intake promotes shift in muscle fiber type composition during sprint interval training in hypoxia | 27 |
| 48 | de Souza et al., 2022 | The acute effect of in natura beetroot juice intake on intra-session exercise sequences during concurrent training | 20 |
| 49 | Dominguez 2017 | Effects of beetroot juice supplementation on a 30-s high-intensity inertial cycle ergometer test | 15 |
| 50 | Dumar 2021 | Acute Beetroot Juice Supplementation Attenuates Morning-Associated Decrements in Supramaximal Exercise Performance in Trained Sprinters | 10 |
| 51 | Engan et al., 2012 | Acute dietary nitrate supplementation improves dry static apnea performance | 20 |
| 52 | Esen 2019 | No Effect of Beetroot Juice Supplementation on 100-m and 200-m Swimming Performance in Moderately Trained Swimmers | 10 |
| 53 | Esen 2022 | Acute Beetroot Juice Supplementation Enhances Intermittent Running Performance but Does Not Reduce Oxygen Cost of Exercise among Recreational Adults | 12 |
| 54 | Fan 2018 | Oral Nitrate Supplementation Differentially Modulates Cerebral Artery Blood Velocity and Prefrontal Tissue Oxygenation During 15 km Time-Trial Cycling in Normoxia but Not in Hypoxia | 12 |
| 55 | Fernandez-Elias 2020 | Acute beetroot juice supplementation does not improve match-play activity in professional tennis players. | 9 |
| 56 | Finkel et al., 2018 | Long-term effects of NO3– on the relationship between oxygen uptake and power after three weeks of supplemented HIHVT | 17 |
| 57 | Flanagan et al., 2016 | The effects of nitrate-rich supplementation on neuromuscular efficiency during heavy resistance exercise | 14 |
| 58 | Flueck 2019 | Influence of Equimolar Doses of Beetroot Juice and Sodium Nitrate on Time Trial Performance in Handcycling | 14 |
| 59 | Fowler et al., 2020 | No thermoregulatory or ergogenic effect of dietary nitrate among physically inactive males, exercising above gas exchange threshold in hot and dry conditions | 11 |
| 60 | Fulford 2013 | Influence of dietary nitrate supplementation on human skeletal muscle metabolism and force production during maximum voluntary contractions | 8 |
| 61 | Gallardo et al., 2021 | Dose-response effect of dietary nitrate on muscle contractility and blood pressure in older subjects: a pilot study | 9 |
| 62 | Garnacho-Castano 2015 | Effects of a single dose of beetroot juice on cycling time trial performance at ventilatory thresholds intensity in male triathletes | 12 |
| 63 | Garnacho-Castano 2020 | Understanding the effects of beetroot juice intake on CrossFit performance by assessing hormonal, metabolic and mechanical response: A randomized, double-blind, crossover design. | 12 |
| 64 | Garnacho-Castaño et al., 2022 | Circulating nitrate-nitrite reduces oxygen uptake for improving resistance exercise performance after rest time in well-trained crossfit athletes | 11 |
| 65 | Gasier 2017 | Effects of oral sodium nitrate on forearm blood flow, oxygenation and exercise performance during acute exposure to hypobaric hypoxia (4300 m) | 10 |
| 66 | Ghiarone 2017 | Effect of acute nitrate ingestion on VO2 response at different exercise intensity domains | 11 |
| 67 | Gholami 2019 | High doses of sodium nitrate prior to exhaustive exercise increases plasma peroxynitrite levels in well-trained subjects: randomized, double-blinded, crossover study | 10 |
| 68 | Glaister 2015 | Effects of dietary nitrate, caffeine, and their combination on 20-km cycling time trial performance | 14 |
| 69 | Gonzalez 2019 | Red Spinach Extract Supplementation Improves Cycle Time Trial Performance in Recreationally Active Men and Women | 17 |
| 70 | Haider 2014 | Nitrate supplementation enhances the contractile properties of human skeletal muscle. | 19 |
| 71 | Handzlik 2013 | Likely additive ergogenic effects of combined preexercise dietary nitrate and caffeine ingestion in trained cyclists | 14 |
| 72 | Hoon 2014(a) | The effect of variable doses of inorganic nitrate-rich beetroot juice on simulated 2,000-m rowing performance in trained athletes | 10 |
| 73 | Hoon 2014(b) | Nitrate supplementation and high-intensity performance in competitive cyclists | 26 |
| 74 | Hoon 2015 | The effect of nitrate supplementation on muscle contraction in healthy adults | 18 |
| 75 | Horiuchi 2017 | Muscle oxygenation profiles between active and inactive muscles with nitrate supplementation under hypoxic exercise | 9 |
| 76 | Hurst 2020 | No Differences Between Beetroot Juice and Placebo on Competitive 5-km Running Performance: A Double-Blind, Placebo-Controlled Trial | 70 |
| 77 | Husmann 2019 | Dietary nitrate supplementation improves exercise tolerance by reducing muscle fatigue and perceptual responses | 12 |
| 78 | Jo 2017 | The Effects of Multi-Day vs. Single Pre-exercise Nitrate Supplement Dosing on Simulated Cycling Time Trial Performance and Skeletal Muscle Oxygenation | 29 |
| 79 | Jodra 2020 | Effect of Beetroot Juice Supplementation on Mood, Perceived Exertion, and Performance during a 30-Second Wingate Test | 15 |
| 80 | Jonvik 2018(a) | Repeated-sprint performance and plasma responses following beetroot juice supplementation do not differ between recreational, competitive and elite sprint athletes | 52 |
| 81 | Jonvik 2018(b) | The effect of beetroot juice supplementation on dynamic apnea and intermittent sprint performance in elite female water polo players | 14 |
| 82 | Jonvik et al., 2019 | Sucrose but Not Nitrate Ingestion Reduces Strenuous Cycling-induced Intestinal Injury | 16 |
| 83 | Jonvik et al., 2020 | The impact of beetroot juice supplementation on muscle endurance, maximal strength, and countermovement jump performance | 15 |
| 84 | Kell 2013 | Effects of nitrate on the power–duration relationship for severe-intensity exercise | 9 |
| 85 | Kelly 2014 | Dietary nitrate supplementation: effects on plasma nitrite and pulmonary O2 uptake dynamics during exercise in hypoxia and normoxia | 12 |
| 86 | Kelly et al., 2013 | Effects of short-term dietary nitrate supplementation on blood pressure, O2 uptake kinetics, and muscle and cognitive function in older adults | 12 |
| 87 | Kent 2018(a) | Dietary nitrate supplementation does not improve cycling time-trial performance in the heat | 12 |
| 88 | Kent 2018(b) | Effect of dietary nitrate supplementation on thermoregulatory and cardiovascular responses to submaximal cycling in the heat | 12 |
| 89 | Kent 2019 | The effect of beetroot juice supplementation on repeat-sprint performance in hypoxia | 12 |
| 90 | Khosravi et al., 2020 | The effect of beetroot juice supplementation on muscle performance during isokinetic knee extensions in male taekwondo athletes | 12 |
| 91 | Kokkinoplitis 2014 | The effect of beetroot juice on repeated sprint performance and muscle force production | 7 |
| 92 | Kramer 2016 | The effect of six days of dietary nitrate supplementation on performance in trained CrossFit athletes | 12 |
| 93 | Lane 2014 | Single and combined effects of beetroot juice and caffeine supplementation on cycling time trial performance | 24 |
| 94 | Lansley 2011(a) | Acute dietary nitrate supplementation improves cycling time trial performance | 9 |
| 95 | Lansley 2011(b) | Dietary nitrate supplementation reduces the O2 cost of walking and running: a placebo-controlled study | 9 |
| 96 | Lara 2015 | Effects of handgrip exercise or inorganic nitrate supplementation on 24-h ambulatory blood pressure and peripheral arterial function in overweight and obese middle age and older adults: A pilot RCT | 30 |
| 97 | Larsen 2007 | Effect of dietary nitrate on oxygen cost during exercise | 9 |
| 98 | Larsen 2010 | Dietary nitrate reduces maximal oxygen consumption while maintaining work performance in maximal exercise | 9 |
| 99 | Larsen 2011 | Dietary inorganic nitrate improves mitochondrial efficiency in humans | 14 |
| 100 | Le 2019 | Effects of acute nitric oxide precursor intake on peripheral and central fatigue during knee extensions in healthy men | 15 |
| 101 | Lee 2019 | Acute beetroot juice supplementation does not attenuate knee extensor exercise muscle fatigue in a healthy young population | 35 |
| 102 | Liubertas et al., 2020 | The influence of amaranth (Amaranthus hypochondriacus) dietary nitrates on the aerobic capacity of physically active young persons | 13 |
| 103 | Lopez-Samanes 2020(a) | Does Acute Beetroot Juice Supplementation Improve ~ Neuromuscular Performance and Match Activity in Young Basketball Players? A Randomized, Placebo-Controlled Study. | 10 |
| 104 | Lopez-Samanes 2020(b) | Effects of beetroot juice ingestion on physical performance in highly competitive tennis players | 13 |
| 105 | Lowings 2017 | Effect of dietary nitrate supplementation on swimming performance in trained swimmers | 10 |
| 106 | MacLeod 2015 | Acute beetroot juice supplementation does not improve cycling performance in normoxia or moderate hypoxia | 11 |
| 107 | Martin 2014 | No improvement of repeated-sprint performance with dietary nitrate | 16 |
| 108 | Masschelein 2012 | Dietary nitrate improves muscle but not cerebral oxygenation status during exercise in hypoxia | 15 |
| 109 | McIlvenna et al., 2019 | Lower limb ischemic preconditioning combined with dietary nitrate supplementation does not influence time-trial performance in well-trained cyclists | 10 |
| 110 | McQuillan 2017(a) | Dietary nitrate fails to improve 1 and 4 km cycling performance in highly trained cyclists | 9 |
| 111 | McQuillan 2017(b) | The Efect of Dietary Nitrate Supplementation on Physiology and Performance in Trained Cyclists | 8 |
| 112 | McQuillan 2018 | The effect of nitrate supplementation on cycling performance in the heat in well-trained cyclists | 8 |
| 113 | Meamarbashi 2014 | Moderate dose of watercress and red radish does not reduce oxygen consumption during graded exhaustive exercise | 36 |
| 114 | Montenegro 2017 | Betalain-rich concentrate supplementation improves exercise performance and recovery in competitive triathletes | 22 |
| 115 | Moore 2017 | Red Spinach Extract Increases Ventilatory Threshold during Graded Exercise Testing | 15 |
| 116 | Mosher 2016 | Ingestion of a nitric oxide enhancing supplement improves resistance exercise performance | 12 |
| 117 | Mosher 2019 | High dose Nitrate ingestion does not improve 40 km cycling time trial performance in trained cyclists | 11 |
| 118 | Muggeridge 2013 | The effects of a single dose of concentrated beetroot juice on performance in trained flatwater kayakers | 8 |
| 119 | Muggeridge 2014 | A single dose of beetroot juice enhances cycling performance in simulated altitude | 9 |
| 120 | Muggeridge 2015 | Acute whole body UVA irradiation combined with nitrate ingestion enhances time trial performance in trained cyclists | 9 |
| 121 | Muggeridge et al., 2016 | The efects of dietary nitrate supplementation on the adaptations to sprint interval training in previously untrained males | 27 |
| 122 | Mumford 2018 | Effect of 1-week betalain-rich beetroot concentrate supplementation on cycling performance and select physiological parameters | 28 |
| 123 | Murphy 2012 | Whole beetroot consumption acutely improves running performance | 11 |
| 124 | Nyakayiru 2017 | Beetroot juice supplementation improves high-intensity intermittent type exercise performance in trained soccer players | 32 |
| 125 | Nyakayiru JM 2017 | No effect of acute and 6-day nitrate supplementation on VO2 and time-trial performance in highly trained cyclists | 17 |
| 126 | Nyback 2017 | Physiological and performance effects of nitrate supplementation during roller-skiing in normoxia and normobaric hypoxia | 8 |
| 127 | Oskarsson 2018 | No individual or combined effects of caffeine and beetroot-juice supplementation during submaximal or maximal running | 9 |
| 128 | Papadopoulos 2018 | Beetroot increases muscle performance and oxygenation during sustained isometric exercise, but does not alter muscle oxidative efficiency and microvascular reactivity at rest. | 16 |
| 129 | Pawlak 2019 | Beetroot juice does not enhance supramaximal intermittent exercise performance in elite endurance athletes | 11 |
| 130 | Peacock 2012 | Dietary nitrate does not enhance running performance in elite cross-country skiers | 10 |
| 131 | Peeling 2015 | Beetroot juice improves on-water 500 m time-trial performance, and laboratory-based paddling economy in national and international-level kayak athletes | 11 |
| 132 | Perez 2019 | The Effects of Beetroot Juice on VO2max and Blood Pressure during Submaximal Exercise | 20 |
| 133 | Pinna 2014 | Efect of beetroot juice supplementation on aerobic response during swimming | 14 |
| 134 | Porcelli 2015 | Aerobic fitness affects the exercise performance responses to nitrate supplementation | 21 |
| 135 | Porcelli 2016 | Effects of a short-term high-nitrate diet on exercise performance | 7 |
| 136 | Pryor 2017 | The Effect of Betaine on Nitrate and Cardiovascular Response to Exercise | 10 |
| 137 | Puype 2015 | No effect of dietary nitrate supplementation on endurance training in hypoxia | 22 |
| 138 | Ranchal-Sanchez 2020 | Acute effects of beetroot juice supplements on resistance training: A randomized double-blind crossover. | 11 |
| 139 | Reynolds 2020 | Acute Ingestion of Beetroot Juice Does Not Improve Short-Duration Repeated Sprint Running Performance in Male Team Sport Athletes. | 16 |
| 140 | Richard et al., 2018 | Time-Trial Performance in World-Class Speed Skaters After Chronic Nitrate Ingestion | 9 |
| 141 | Rienks 2015 | Effect of beetroot juice on moderate-intensity exercise at a constant rating of perceived exertion | 10 |
| 142 | Rimer 2016(a) | Increase in maximal cycling power with acute dietary nitrate supplementation | 13 |
| 143 | Robinson 2020 | Influence of Dietary Nitrate Supplementation on High-Intensity Intermittent Running Performance at Different Doses of Normobaric Hypoxia in Endurance-Trained Males | 8 |
| 144 | Rodríguez-Fernández et al., 2021 | Beetroot juice supplementation increases concentric and eccentric muscle power output | 18 |
| 145 | Roelofs 2017 | Effects of Pomegranate Extract on Blood Flow and Vessel Diameter after High-Intensity Exercise in Young, Healthy Adults. | 19 |
| 146 | Rokkedal-Lausch 2019 | Chronic high-dose beetroot juice supplementation improves time trial performance of well-trained cyclists in normoxia and hypoxia | 12 |
| 147 | Rossetti 2017 | Dietary nitrate supplementation increases acute mountain sickness severity and sense of effort during hypoxic exercise | 20 |
| 148 | Rothwell & Alkhatib, 2014 | Effects of acute dietary nitrate supplementation on 30-second Wingate performance in healthy collegiate males | 20 |
| 149 | Sandbakk 2015 | Effects of acute supplementation of l-arginine and nitrate on endurance and sprint performance in elite athletes | 9 |
| 150 | Santana 2019 | Nitrate Supplementation Combined with a Running Training Program Improved Time-Trial Performance in Recreationally Trained Runners | 16 |
| 151 | Shannon 2016 | Dietary nitrate supplementation enhances high-intensity running performance in moderate normobaric hypoxia, independent of aerobic fitness | 12 |
| 152 | Shannon 2017 (a) | Dietary nitrate supplementation enhances short but not longer duration running time-trial performance | 8 |
| 153 | Shannon 2017 (b) | Effects of dietary nitrate supplementation on physiological responses, cognitive function, and exercise performance at moderate and very-high simulated altitude | 10 |
| 154 | Siervo 2016 | Dietary nitrate does not affect physical activity or outcomes in healthy older adults in a randomized, crossover trial. | 20 |
| 155 | Smith 2019 | An acute dose of inorganic dietary nitrate does not improve high-intensity, intermittent exercise performance in temperate or hot and humid conditions | 12 |
| 156 | Sousa et al., 2022 | Dietary nitrate supplementation is not helpful for endurance performance at simulated altitude even when combined with intermittent normobaric hypoxic training | 30 |
| 157 | Tan 2018 | Beetroot juice ingestion during prolonged moderate-intensity exercise attenuates progressive rise in O2 uptake | 12 |
| 158 | Tan et al., 2022 | Effects of dietary nitrate supplementation on performance and muscle oxygenation during resistance exercise in men | 14 |
| 159 | Tatlici, 2021 | The effects of acute beetroot juice supplementation on lower and upper body isokinetic strength of the wrestlers | 8 |
| 160 | Thompson 2014 | Influence of dietary nitrate supplementation on physiological and cognitive responses to incremental cycle exercise | 16 |
| 161 | Thompson 2015 | Dietary nitrate improves sprint performance and cognitive function during prolonged intermittent exercise | 16 |
| 162 | Thompson 2016 | Dietary nitrate supplementation improves sprint and high-intensity intermittent running performance | 36 |
| 163 | Thompson 2017 | Influence of dietary nitrate supplementation on physiological and muscle metabolic adaptations to sprint interval training | 36 |
| 164 | Thompson 2018 | Discrete physiological effects of beetroot juice and potassium nitrate supplementation following 4-wk sprint interval training | 30 |
| 165 | Tillin 2018 | Nitrate supplement benefits contractile forces in fatigued but not unfatigued muscle | 17 |
| 166 | Torregrosa-Garcia 2019 | Pomegranate Extract Improves Maximal Performance of Trained Cyclists after an Exhausting Endurance Trial: A Randomised Controlled Trial | 26 |
| 167 | Trexler 2014 | Effects of pomegranate extract on blood flow and running time to exhaustion | 19 |
| 168 | Trexler et al., 2020 | Effects of citrulline malate and beetroot juice supplementation on energy metabolism and blood flow during submaximal resistance exercise | 27 |
| 169 | Van 2016 | Betalain-rich concentrate supplementation improves exercise performance in competitive runners | 22 |
| 170 | Vanhatalo 2010 | Acute and chronic effects of dietary nitrate supplementation on blood pressure and the physiological responses to moderate-intensity and incremental exercise | 8 |
| 171 | Vanhatalo 2011 | Dietary nitrate reduces muscle metabolic perturbation and improves exercise tolerance in hypoxia | 9 |
| 172 | Vasconcellos 2017 | A single dose of beetroot gel rich in nitrate does not improve performance but lowers blood glucose in physically active individuals | 25 |
| 173 | Whitfield 2017 | Beetroot juice increases human muscle force without changing Ca2þ-handling proteins | 8 |
| 174 | Wickham 2019 | No effect of beetroot juice supplementation on exercise economy and performance in recreationally active females despite increased torque production | 12 |
| 175 | Wilkerson 2012 | Influence of acute dietary nitrate supplementation on 50 mile time trial performance in well-trained cyclists | 8 |
| 176 | Williams 2020 | Effect of acute beetroot juice supplementation on bench press power, velocity, and repetition volume. | 11 |
| 177 | Wylie 2013(a) | Beetroot juice and exercise: pharmacodynamic and dose–response relationships | 10 |
| 178 | Wylie 2013(b) | Dietary nitrate supplementation improves team sport-specific intense intermittent exercise performance | 14 |
| 179 | Wylie 2016 | Influence of beetroot juice supplementation on intermittent exercise performance | 10 |
| 180 | Wylie 2019 | Human skeletal muscle nitrate store: influence of dietary nitrate supplementation and exercise | 13 |
