# Supplementary material for: Dietary Nitrate Supplementation and Exercise Performance: An Umbrella Review of 20 Published Systematic Reviews with Meta-analyses
Source: Sports Med. 2025 Mar 14;55(5):1213–31. doi: 10.1007/s40279-025-02194-6 (PMC12106159; doi:10.1007/s40279-025-02194-6)
Supplement: Supplementary file 4 — Supplementary file4 (DOCX 20 KB) [file 40279_2025_2194_MOESM4_ESM.docx]

**Supplementary Table S4** Changes in common measures of aerobic endurance performance comparing NO3^-^ supplementation with placebo-controlled conditions

| **Study** | **No. of studies** | **SMD/MD** | **Mean Change** | **95% CI** | |
| --- | --- | --- | --- | --- | --- |
|  |  |  |  | **Lower Limit** | **Upper Limit** |
| **Outcome: Time-to-exhaustion (TTE)** | | | | | |
| Campos 2018 (long-duration - non-athletes) | 14 | SMD | 0.47 | 0.23 | 0.71 |
| D’Unienville 2021 | 19 | SMD | 0.31 | 0.20 | 0.42 |
| Gao 2021 | 20 | MD (s) | 25.27 | 12.69 | 37.84 |
| Hogwood 2023 | 9 | SMD | 0.08 | -0.21 | 0.37 |
| Hoon 2013 | 3 | SMD | 0.79 | 0.23 | 1.35 |
| McMahon 2017 | 22 | SMD | 0.33 | 0.15 | 0.50 |
| Senefeld 2020 | 32 | SMD | 0.32 | 0.21 | 0.44 |
| Silva 2022 | 50 | SMD | 0.25 | 0.16 | 0.34 |
| **Outcome: Time trial (TT)^#^** | | | | | |
| Campos 2018 (long-duration - non-athletes) | 4 | SMD | 0.12 | -0.37 | 0.61 |
| D’Unienville 2021 | 39 | SMD | 0.12 | 0.07 | 0.16 |
| Gao 2021 | 28 | MD (s) | 1.98 | -0.41 | 4.37 |
| Hoon 2013 | 9 | SMD | 0.11 | -0.16 | 0.37 |
| McMahon 2017 | 28 | SMD | 0.10 | -0.06 | 0.27 |
| Senefeld 2020 | 52 | SMD | 0.09 | 0.00 | 0.17 |
| Silva 2022 | 80 | SMD | 0.05 | -0.02 | 0.11 |
| Silva 2023 | 17 | MD (s) | 2.41 | -10.61 | 15.42 |
| Van De Walle & Vukovich 2018 | 11 | SMD | 0.05 | -0.17 | 0.28 |
| Wong 2022 (TT 5-30 min) | 19 | SMD | 0.15 | 0.00 | 0.31 |
| Wong 2022 (TT 30-60 min) | 6 | SMD | 0.13 | -0.20 | 0.47 |
| **Outcome: Graded exercise test (GXT)** | | | | | |
| Campos 2018 (long-duration - non-athletes) | 5 | SMD | 0.20 | -0.18 | 0.59 |
| D’Unienville 2021 | 15 | SMD | 0.15 | 0.02 | 0.27 |
| Hoon 2013 | 4 | SMD | 0.26 | -0.10 | 0.62 |
| McMahon 2017 | 8 | SMD | 0.25 | -0.06 | 0.56 |
| **Outcome: Total work done** | | | | | |
| Alsharif 2023 | 7 | SMD | 0.06 | -0.13 | 0.26 |
| Gao 2021 | 4 | MD (kJ) | 0.02 | 0.00 | 0.04 |
| **Outcome: Total distance covered** | | | | | |
| Alsharif 2023 | 4 | SMD | 0.17 | 0.09 | 0.24 |
| Gao 2021 | 2 | MD (m) | 163.7 | 18.4 | 309.1 |
| Senefeld 2020 | 6 | SMD | 0.32 | 0.14 | 0.50 |
| Wong 2021 | 3 | SMD | 0.46 | 0.14 | 0.77 |
| **Outcome: Maximal oxygen uptake (**$\dot{\boldsymbol{V}}$**O_2_max)** | | | | | |
| Gao 2021 | 10 | MD (L/min) | 0.04 | -0.02 | 0.10 |
| Hogwood 2023 | 11 | SMD | 0.18 | -0.09 | 0.44 |

CI: Confidence interval; MD: Mean difference; SMD: Standardised mean difference

# a positive value indicates an improvement
