# Supplementary material for: Dietary Nitrate Supplementation and Exercise Performance: An Umbrella Review of 20 Published Systematic Reviews with Meta-analyses
Source: Sports Med. 2025 Mar 14;55(5):1213–31. doi: 10.1007/s40279-025-02194-6 (PMC12106159; doi:10.1007/s40279-025-02194-6)
Supplement: Supplementary file 5 — Supplementary file5 (DOCX 19 KB) [file 40279_2025_2194_MOESM5_ESM.docx]

**Supplementary Table S5** Changes in common measures of muscular fitness and power output performance comparing NO_3_^-^ supplementation with placebo-controlled conditions

| **Study** | **No. of studies** | **SMD/MD** | **Mean Change** | **95% CI** | |
| --- | --- | --- | --- | --- | --- |
|  |  |  |  | **Lower Limit** | **Upper Limit** |
| **Outcome: Muscular strength** | | | | | |
| Alvares 2022 | 26 | SMD | 0.08 | 0.01 | 0.15 |
| Esen 2023 | 11 | SMD | 0.03 | -0.86 | 0.74 |
| Evangelista 2024 | 18 | SMD | 0.64 | 0.25 | 1.03 |
| Lago-Rodríguez 2020 | 5 | SMD | -0.01 | -0.19 | 0.17 |
| **Outcome: Muscular endurance** | | | | | |
| Alvares 2022 | 20 | SMD | 0.31 | 0.16 | 0.46 |
| Evangelista 2024 | 16 | SMD | 0.31 | 0.10 | 0.51 |
| Tan 2023 | 5 | SMD | 0.43 | 0.16 | 0.70 |
| **Outcome: Peak power output (PPO)** | | | | | |
| Alsharif 2023 | 11 | SMD | 0.01 | -0.06 | 0.08 |
| Coggan 2021 | 19 | SMD | 0.45 | 0.3 | 0.61 |
| Esen 2023 | 8 | SMD | 0.25 | 0.01 | 0.5 |
| Tan 2023 | 4 | SMD | 0.2 | 0 | 0.41 |
| Wong 2021 | 10 | SMD | 0.08 | -0.14 | 0.3 |
| **Outcome: Mean power output (MPO)** | | | | | |
| Alsharif 2023 | 13 | SMD | 0.2 | 0.03 | 0.36 |
| Esen 2023 | 6 | SMD | 0.28 | 0.03 | 0.53 |
| Tan 2023 | 3 | SMD | 0.4 | 0.13 | 0.68 |
| Wong 2021 | 8 | SMD | -0.05 | -0.32 | 0.21 |
| **Outcome: Time to reach PPO^#^** | | | | | |
| Alsharif 2023 | 4 | SMD | 0.75 | 0.11 | 1.38 |
| Esen 2023 | 4 | SMD | 0.78 | 0.43 | 1.14 |

CI: Confidence interval; MD: Mean difference; SMD: Standardised mean difference

# a positive value indicates an improvement
