# Supplementary material for: Dietary Nitrate Supplementation and Exercise Performance: An Umbrella Review of 20 Published Systematic Reviews with Meta-analyses
Source: Sports Med. 2025 Mar 14;55(5):1213–31. doi: 10.1007/s40279-025-02194-6 (PMC12106159; doi:10.1007/s40279-025-02194-6)
Supplement: Supplementary file 6 — Supplementary file6 (DOCX 34 KB) [file 40279_2025_2194_MOESM6_ESM.docx]

**Supplementary Table S6** Summary of meta-analyses comparing NO_3_^-^ supplementation with placebo-controlled conditions based on primary studies

| **Outcome** | **k** | **Participants** | **Std. Mean Difference  [95% of CI]** | ***p*** | **Tau^2^** | **Chi^2^ (*p*)** | **I^2^** |
| --- | --- | --- | --- | --- | --- | --- | --- |
| **Time-to-exhaustion (TTE)**  Duration = Acute  Duration = 1-3 days  Duration = > 3 days  Dose= < 6 mmol  Dose= 6−12 mmol  Dose= > 12 mmol | 41  19  5  24  8  16  15 | 1,022  432  102  552  180  353  323 | 0.33 (0.19 to 0.47)  0.15 (−0.04 to 0.35)  0.17 (−0.33 to 0.68)  0.48 (0.28 to 0.69)  0.11 (−0.18 to 0.41)  0.26 (0.05 to 0.47)  0.28 (0.03 to 0.52) | <0.00001  0.13  0.51  <0.0001  0.45  0.02  0.03 | 0.03  0.00  0.12  0.06  0.00  0.00  0.02 | 47.51 (*p* = 0.19)  4.4 (*p* = 1.00)  6.4 (*p* = 0.17*)*  30.8 (*p* = 0.13)  5.1 (*p* = 0.64)  7.1 (*p* = 0.95)  15.6 (*p* = 0.34) | 16%  0%  37%  25%  0%  0%  10% |
| **Time trial (TT)**  Duration = Acute  Duration = 1−3 days  Duration = > 3 days  Dose= < 6 mmol  Dose= 6−12 mmol  Dose= > 12 mmol | 42  35  5  13  8  28  13 | 1,134  824  112  272  136  591  276 | −0.03 (−0.14 to 0.09)  0.00 (−0.14 to 0.14)  0.13 (−0.24 to 0.50)  −0.08 (−0.32 to 0.16)  −0.21 (−0.55 to 0.13)  0.03 (−0.14 to 0.19)  0.02 (−0.21 to 0.26) | 0.65  0.99  0.49  0.50  0.22  0.76  0.84 | 0.00  0.00  0.00  0.00  0.00  0.00  0.00 | 17.59 (*p* = 1.00)  11.7 (*p* = 1.00)  0.2 (*p* = 1.00)  3.5 (*p* = 0.99)  2.3 (*p* = 0.94)  9.8 (*p* = 1.00)  2.2 (*p* = 1.00) | 0%  0%  0%  0%  0%  0%  0% |
| **Graded exercise testing (GXT)**  Duration = Acute  Duration = 1−3 days  Duration = > 3 days  Dose= < 6 mmol  Dose= 6−12 mmol | 11  5  3  5  4  4 | 252  128  64  98  82  106 | 0.18 (−0.07 to 0.42)  0.09 (−0.25 to 0.44)  0.21 (−0.29 to 0.70)  0.23 (−0.17 to 0.63)  0.13 (−0.31 to 0.56)  0.18 (−0.21 to 0.56) | 0.16  0.60  0.41  0.26  0.57  0.37 | 0.00  0.00  0.00  0.00  0.00  0.00 | 1.42 (*p* = 1.00)  0.3 (*p* = 0.99)  0.2 (*p* = 0.91)  0.7 (*p* = 0.95)  0.1 (*p* = 0.99)  0.9 (*p* = 0.83) | 0%  0%  0%  0%  0%  0% |
| **Total work done**  Duration = Acute  Duration = > 3 days  Dose= 6−12 mmol  Dose= > 12 mmol | 10  5  4  4  3 | 232  112  104  100  72 | 0.15 (−0.11 to 0.40)  0.05 (−0.32 to 0.42)  0.20 (−0.18 to 0.59)  0.11 (−0.29 to 0.50)  0.13 (−0.34 to 0.59) | 0.27  0.80  0.30  0.59  0.59 | 0.00  0.00  0.00  0.00  0.00 | 1.3 (*p* = 1.00)  0.3 (*p* = 0.99)  0.3 (*p* = 0.95)  0.7 (*p* = 0.87)  0.2 (*p* = 0.92) | 0%  0%  0%  0%  0% |
| **Total distance covered** | 7 | 250 | 0.42 (0.09 to 0.76) | 0.01 | 0.07 | 9.3 (*p* = 0.16) | 35% |
| **Maximal oxygen uptake (VO_2_max)**  Duration = Acute  Duration = 1−3 days  Duration = > 3 days  Dose= < 6 mmol  Dose= 6−12 mmol  Dose= > 12 mmol | 34  17  4  22  10  18  8 | 884  354  92  536  166  424  202 | −0.10 (−0.26 to 0.05)  −0.06 (−0.27 to 0.15)  −0.08 (−0.49 to 0.33)  −0.01 (−0.18 to 0.16)  −0.08 (−0.38 to 0.23)  0.03 (−0.17 to 0.22)  −0.06 (−0.35 to 0.23) | 0.20  0.59  0.69  0.92  0.61  0.79  0.66 | 0.05  0.00  0.00  0.00  0.00  0.00  0.00 | 44.01 (*p* = 0.10)  1.8 (*p* = 1.00)  1.0 (*p* = 0.80)  17.3 (*p* = 0.69)  1.4 (*p* = 1.00)  7.7 (*p* = 0.97)  2.5 (*p* = 0.92) | 25%  0%  0%  0%  0%  0%  0% |
| **Muscular strength**  Duration = Acute  Duration = 1−3 days  Duration = > 3 days  Dose= < 6 mmol  Dose= 6−12 mmol  Dose= > 12 mmol | 27  18  15  12  15  16  7 | 744  456  364  270  234  410  196 | 0.05 (−0.09 to 0.19)  0.09 (−0.09 to 0.28)  0.06 (−0.15 to 0.27)  0.01 (−0.23 to 0.24)  -0.00 (−0.27 to 0.27)  0.03 (−0.17 to 0.22)  0.17 (−0.12 to 0.45) | 0.50  0.34  0.60  0.97  1.00  0.80  0.25 | 0.00  0.00  0.00  0.00  0.00  2.96  1.51 | 5.27 (*p* = 1.00)  4.7 (*p* = 1.00)  1.5 (*p* = 1.00)  0.9 (*p* = 1.00)  0.8 (*p* = 1.00)  3.0 (*p* = 1.00)  1.5 (*p* = 0.96) | 0%  0%  0%  0%  0%  0%  0% |
| **Muscular endurance**  Duration = Acute  Duration = > 3 days  Dose= 6−12 mmol  Dose= > 12 mmol | 22  15  9  14  9 | 570  370  190  356  234 | 0.48 (0.23 to 0.74)  0.44 (0.12 to 0.77)  0.50 (0.13 to 0.87)  0.49 (0.11 to 0.87)  0.32 (0.06 to 0.58) | 0.0002  0.008  0.009  0.01  0.02 | 0.20  0.24  0.11  0.33  0.00 | 46.34 (*p* = 0.001)  33.3 (*p* = 0.003)  12.2 (*p* = 0.14)  37.4 (*p* = 0.0004)  7.2 (*p* = 0.52) | 55%  58%  35%  65%  0% |
| **Peak power output (PPO)**  Duration = Acute  Duration = > 3 days  Dose= < 6 mmol  Dose= 6−12 mmol  Dose= > 12 mmol | 27  18  9  3  18  5 | 742  437  294  78  422  218 | 0.25 (0.10 to 0.39)  0.31 (0.12 to 0.50)  0.19 (−0.04 to 0.42)  0.27 (−0.18 to 0.72)  0.18 (−0.02 to 0.37)  0.34 (0.05 to 0.63) | 0.0008  0.001  0.10  0.24  0.07  0.02 | 0.00  0.00  0.00  0.00  0.00  0.01 | 18.81 (*p* = 0.84)  15.0 (*p* = 0.59)  2.7 (*p* = 0.95)  1.0 (*p* = 0.60)  11.5 (*p* = 0.83)  4.4 (*p* = 0.36) | 0%  0%  0%  0%  0%  9% |
| **Mean power output (MPO)**  Duration = Acute  Duration = 1−3 days  Duration = > 3 days  Dose= < 6 mmol  Dose= 6−12 mmol | 18  8  6  6  4  14 | 522  226  128  214  102  326 | 0.14 (−0.03 to 0.32)  0.27 (0.01 to 0.54)  −0.02 (−0.36 to 0.33)  0.07 (−0.20 to 0.34)  0.13 (−0.26 to 0.52)  0.07 (−0.15 to 0.29) | 0.10  0.04  0.93  0.61  0.51  0.52 | 0.00  0.00  0.00  0.00  0.00  0.00 | 5.39 (*p* = 1.00)  3.1 (*p* = 0.87)  0.2 (*p* = 1.00)  0.4 (*p* = 0.99)  0.5 (*p* = 0.93)  2.3 (*p* = 1.00) | 0%  0%  0%  0%  0%  0% |
| **Time to PPO** | 4 | 194 | −0.76 (−1.18 to −0.33) | 0.0005 | 0.08 | 5.2 (*p* = 0.16) | 43% |
