# Supplementary material for: Dietary Nitrate Supplementation and Exercise Performance: An Umbrella Review of 20 Published Systematic Reviews with Meta-analyses
Source: Sports Med. 2025 Mar 14;55(5):1213–31. doi: 10.1007/s40279-025-02194-6 (PMC12106159; doi:10.1007/s40279-025-02194-6)

**Supplementary Figure S7** Forest plots of the effect of NO3- supplementation on each performance outcome

Forest plot of time-to-exhaustion (TTE)


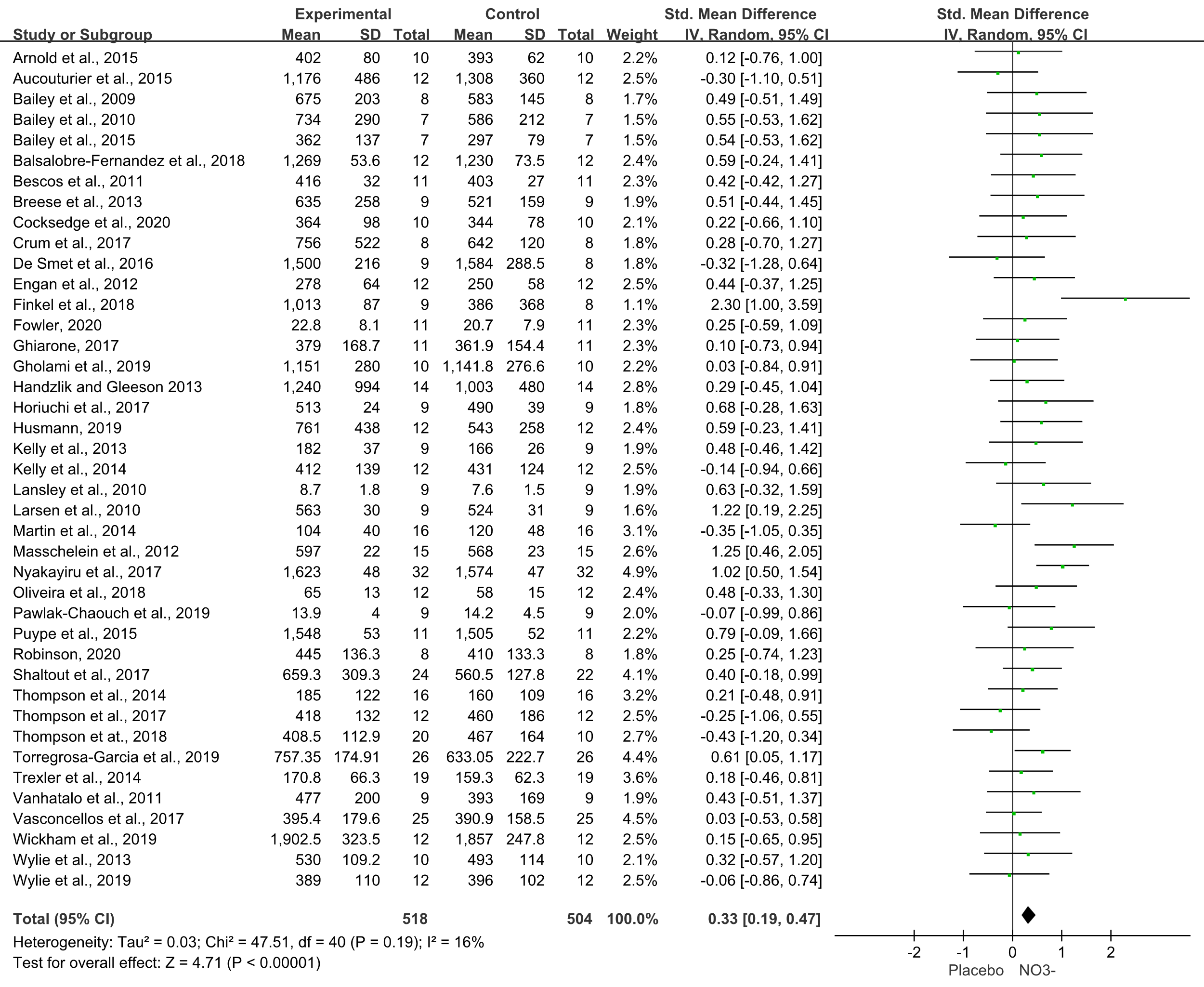


Forest plot of time trial (TT)


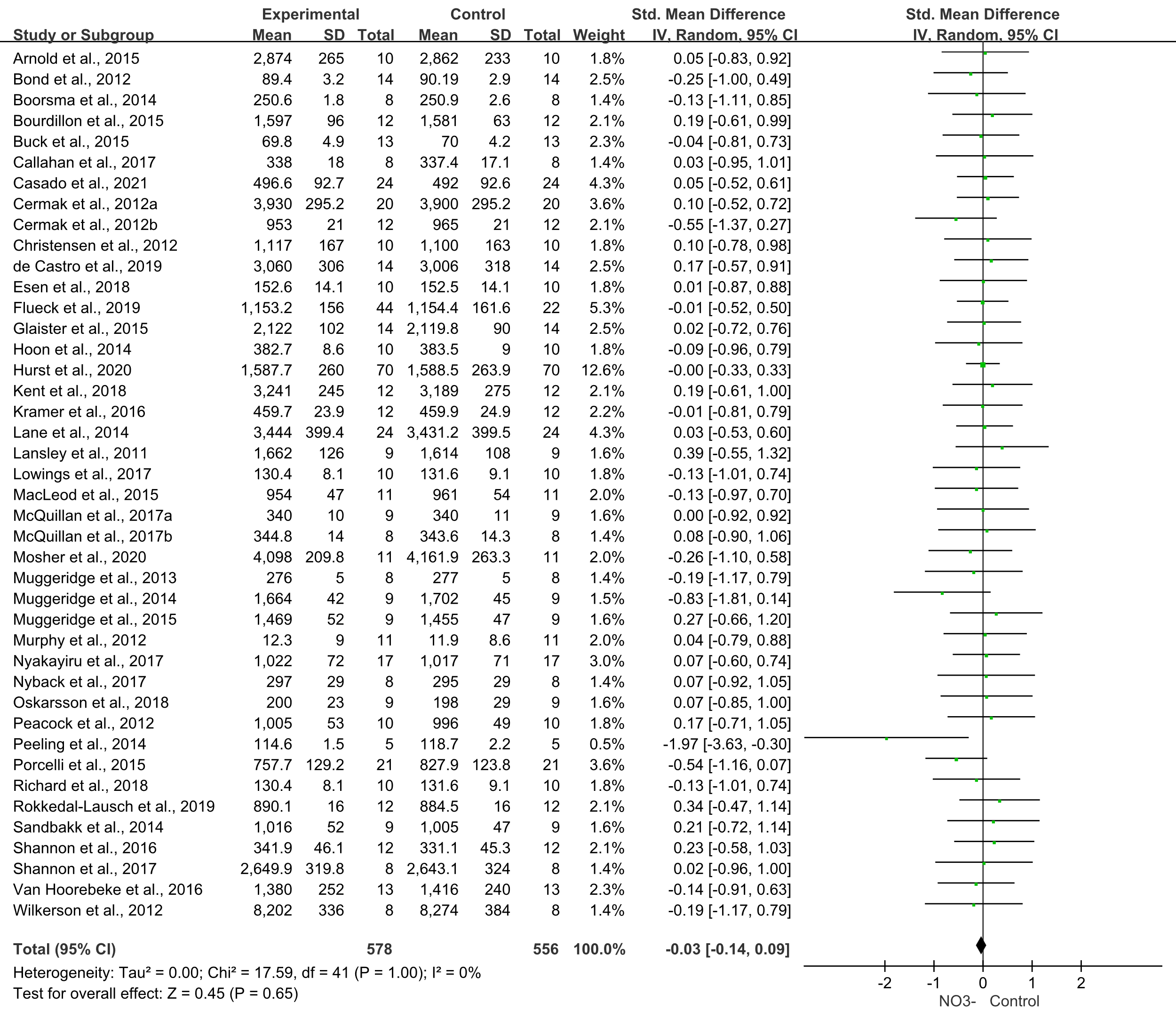


Forest plot of graded exercise test (GXT)


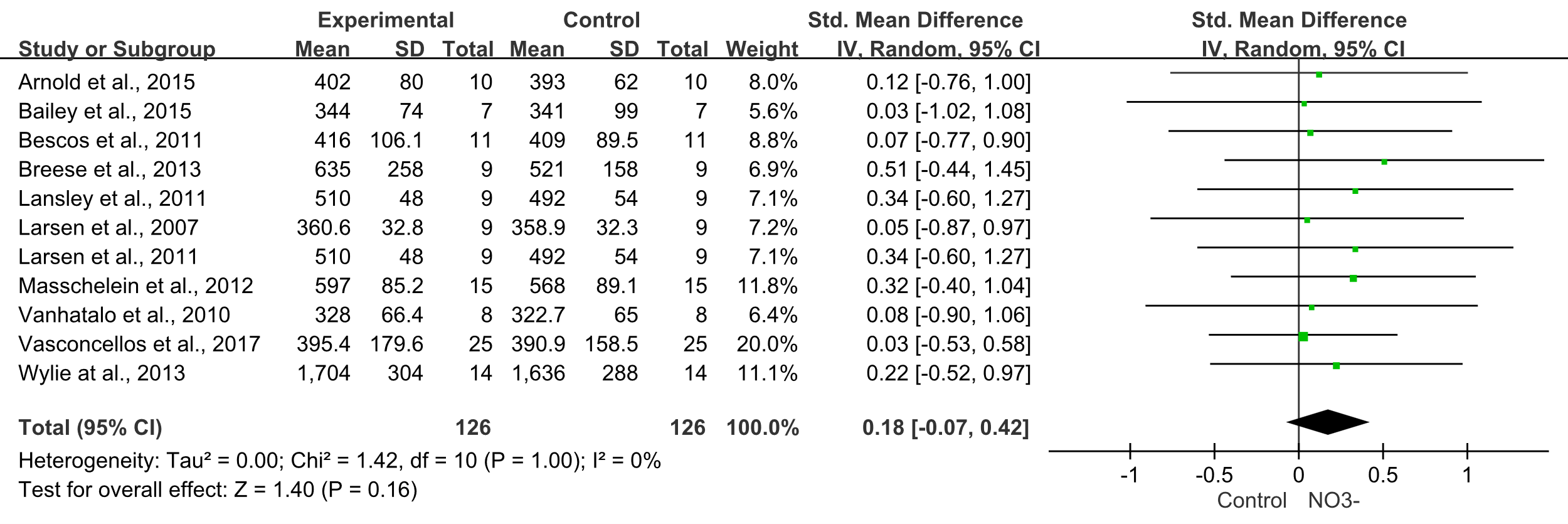


Forest plot of total work done


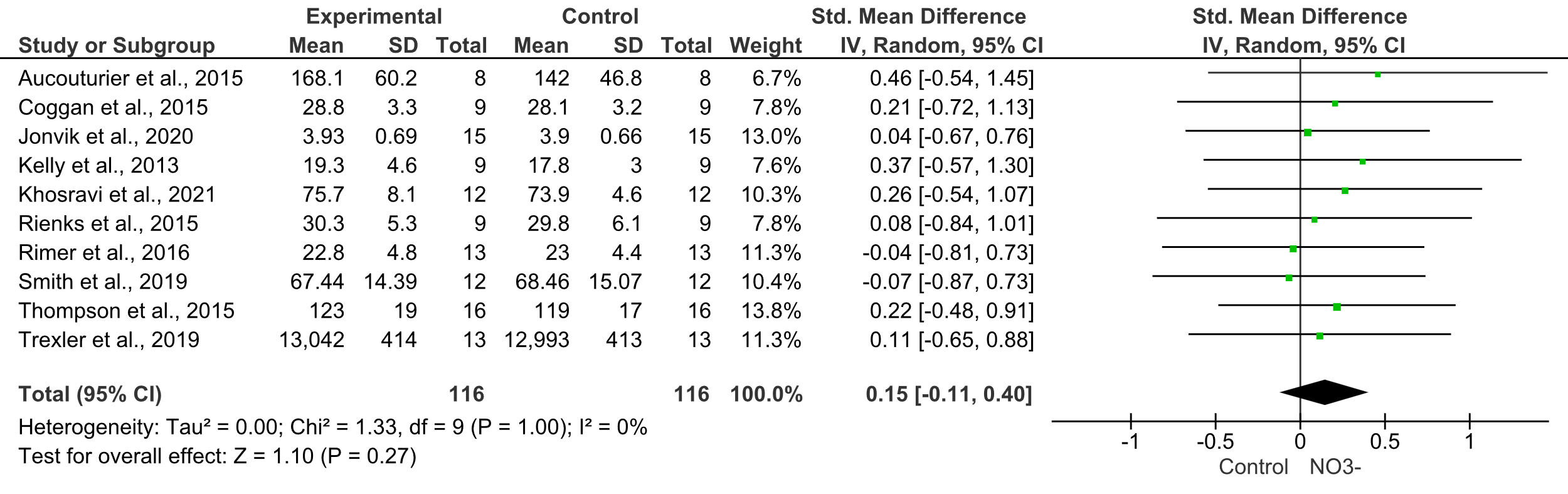


Forest plot of total distance covered


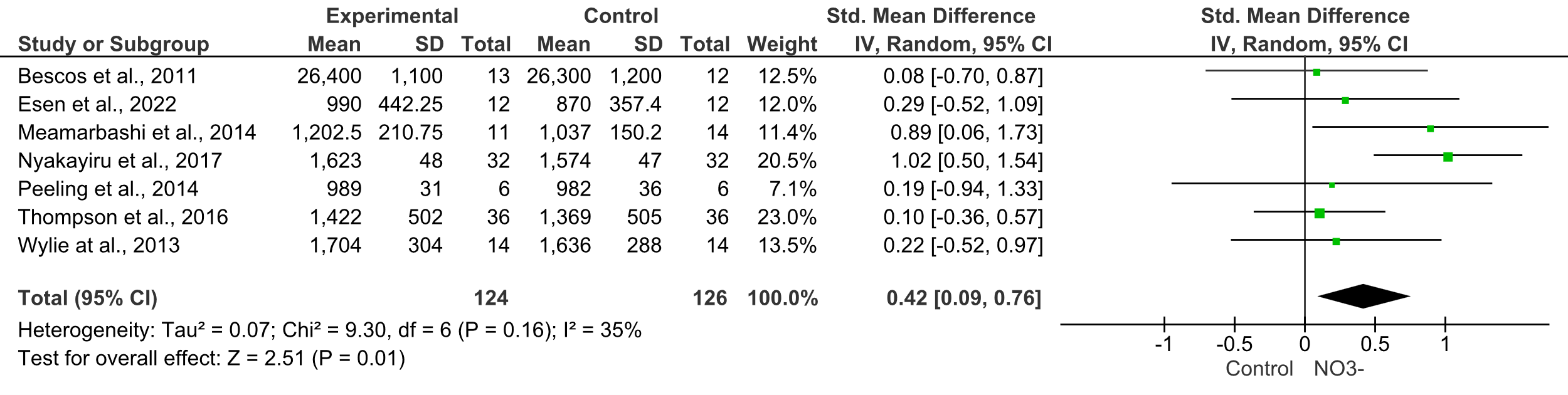


Forest plot of maximal oxygen uptake (VO_2_max)


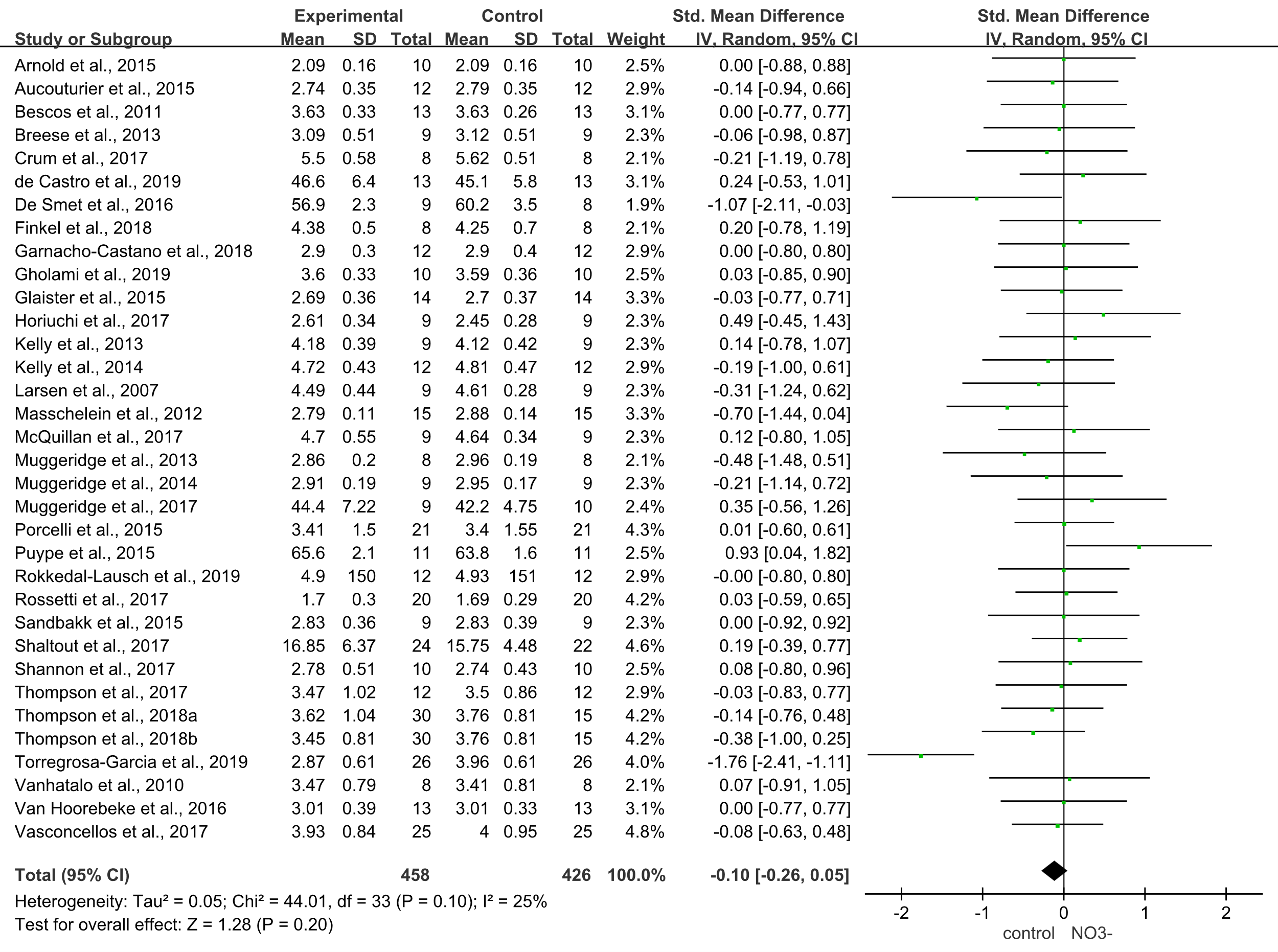


Forest plot of muscular strength


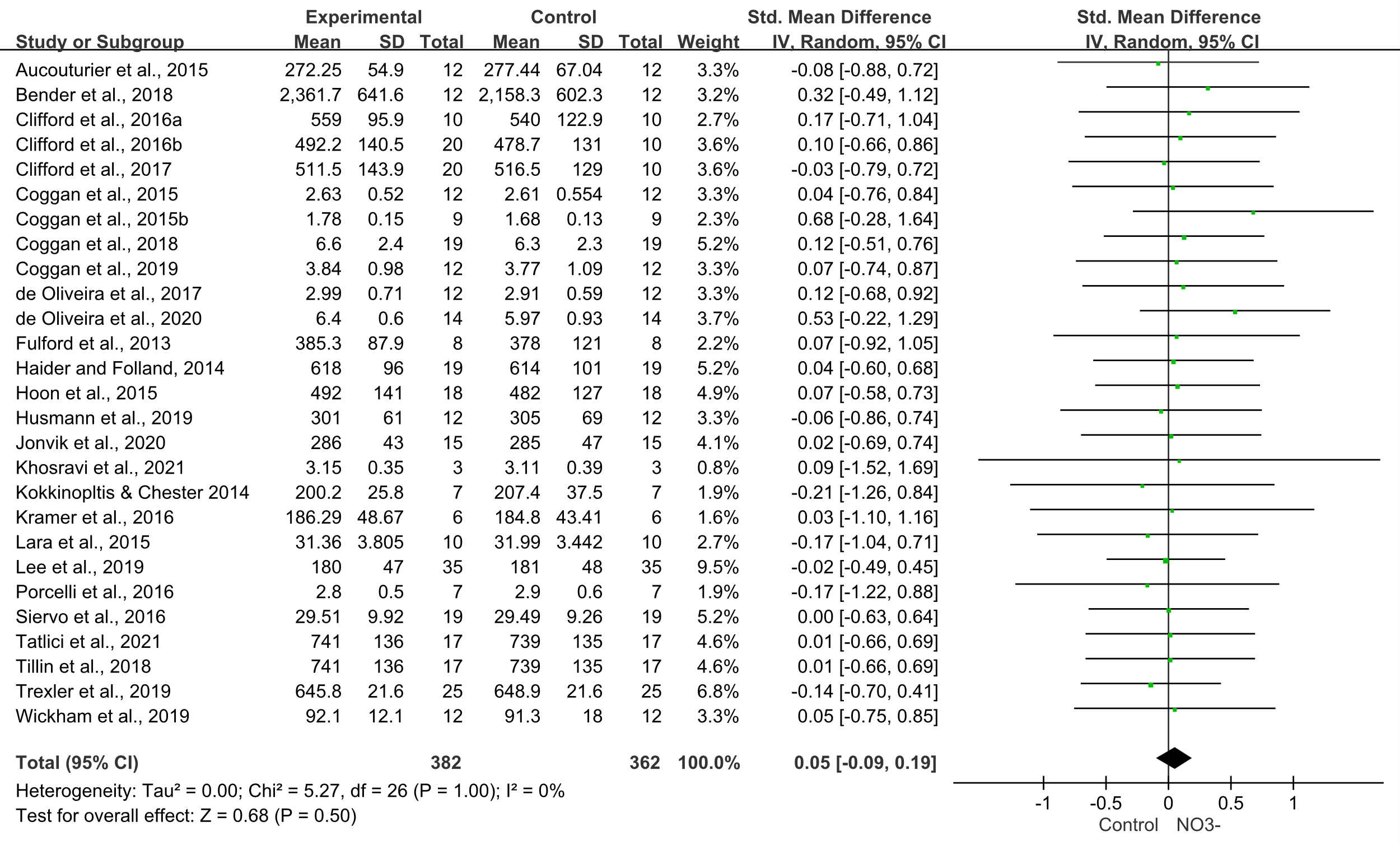


Forest plot of muscular endurance


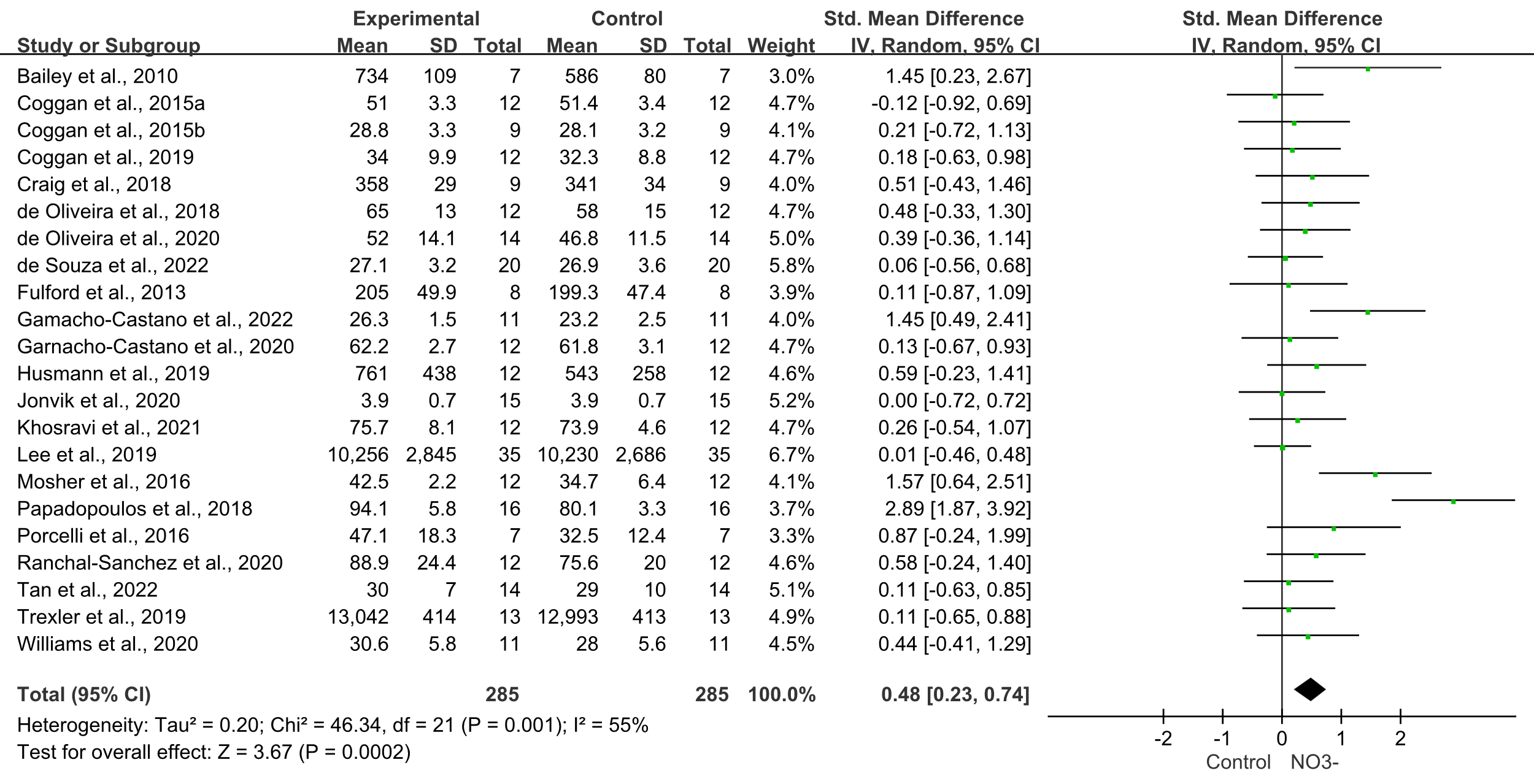


Forest plot of peak power output (PPO)


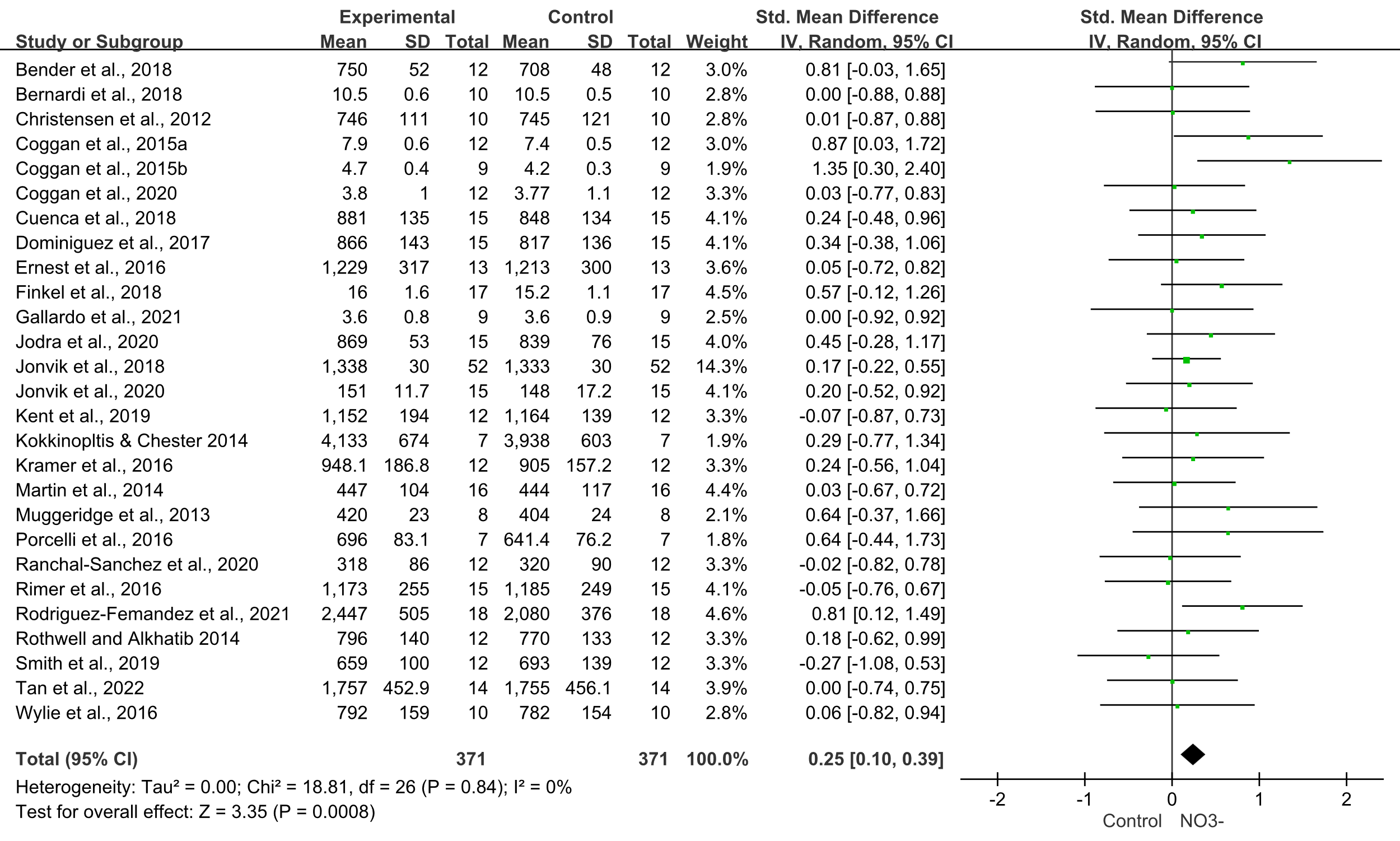


Forest plot of mean power output (MPO)


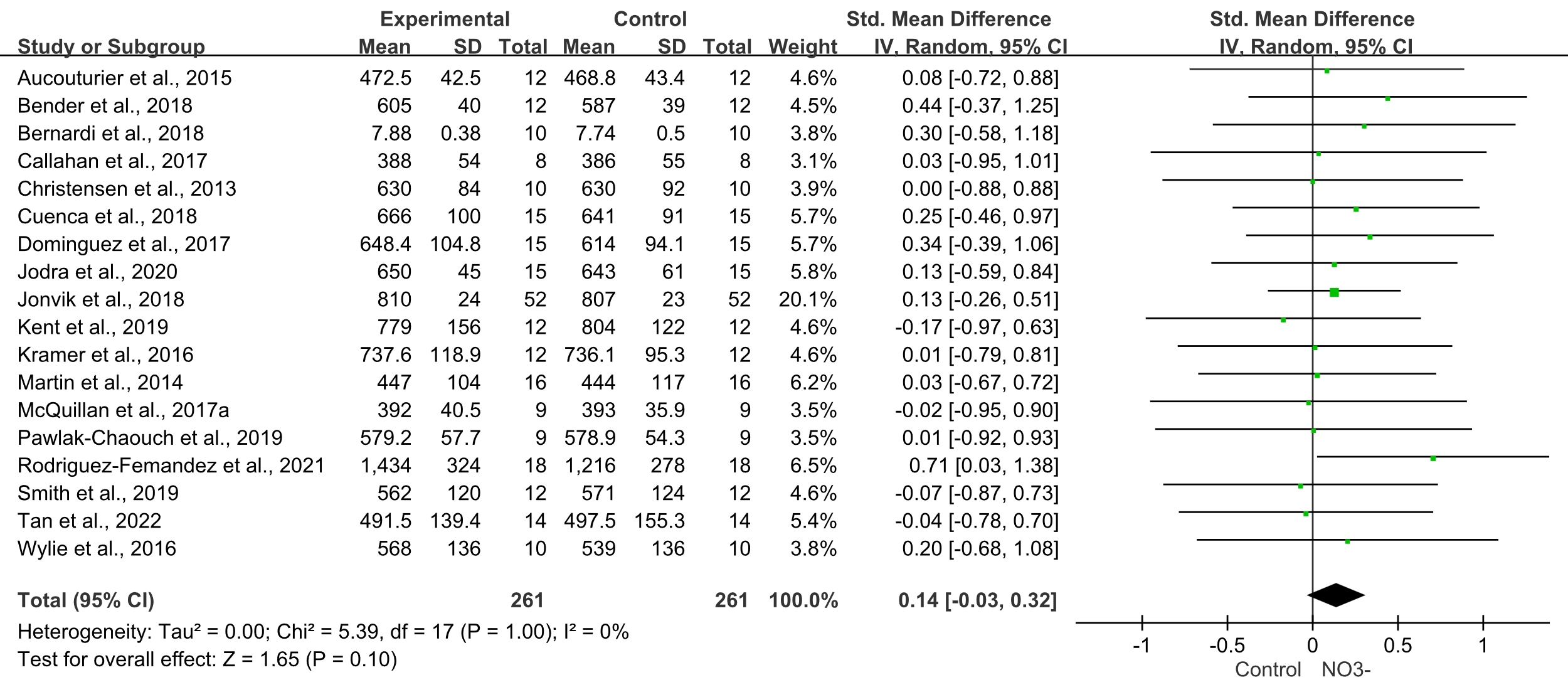


Forest plot of time to PPO
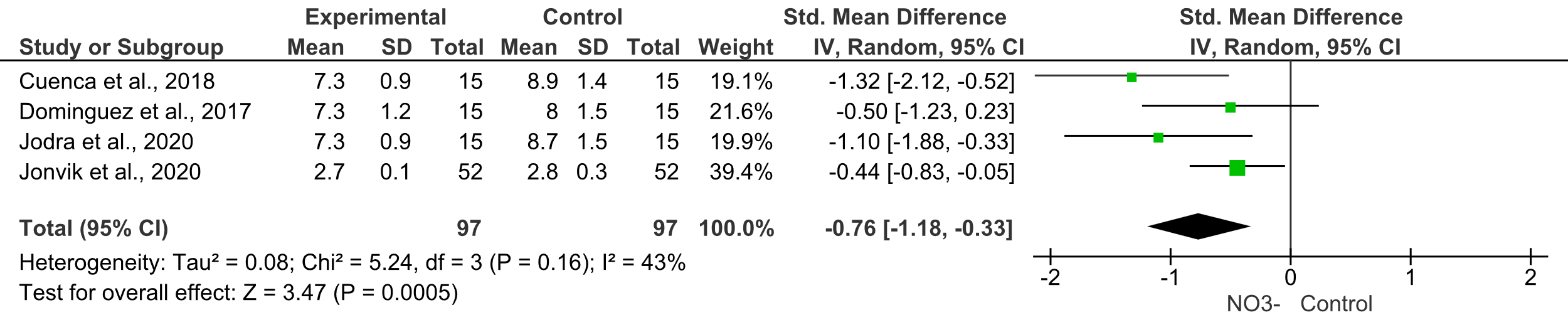

Supplement: Supplementary file 7 — Supplementary file7 (DOCX 14426 KB) [file 40279_2025_2194_MOESM7_ESM.docx]
